# Genomic surveillance of carbapenem-resistant *Acinetobacter baumannii* from bloodstream infections in Thailand reveals widespread dissemination of *bla*_NDM_–harboring mobile genetic elements

**DOI:** 10.1371/journal.pone.0353488

**Published:** 2026-07-13

**Authors:** Aekkawat Unahalekhaka, Kulsumpun Krobanan, Watcharaporn Kamjumphol, Phimrata Leethongdee, Yanisa Sukheephak, Patchaya Boonjan, Kasidet Thapaan, Wiphat Klayut, Orapan Sripichai, Nalumon Thadtapong, Chattip Kurehong, Kranokpron Moolwang, Donlaya Muanplueng, Warawan Wongboot, Pilailuk Akkapaiboon Okada, Jiraphan Premsuriya

**Affiliations:** 1 National Antimicrobial Resistance Surveillance Center, Thailand (NARST), National Institute of Health, Department of Medical Sciences, Ministry of Public Health, Nonthaburi, Thailand; 2 National Institute of Health, Department of Medical Sciences, Ministry of Public Health, Nonthaburi, Thailand; 3 Princess Srisavangavadhana Faculty of Medicine, Chulabhorn Royal Academy, Bangkok, Thailand; 4 Research Center on Clinical and System Microbiology (RCSyM), Chulabhorn Royal Academy, Bangkok, Thailand; Faculty of Veterinary Medicine, Qena University, EGYPT

## Abstract

Carbapenem-resistant *Acinetobacter baumannii* (CRAB) is a major cause of hospital-acquired bloodstream infections in Thailand, with limited treatment options and a strong association with high morbidity and mortality. We performed nationwide genomic surveillance of 41 CRAB isolates from bloodstream infections collected across Thailand between 2020 and 2024 to characterize the population structure, antimicrobial resistance determinants, and the genomic context of *bla*_NDM_-harboring mobile genetic elements (MGEs). Whole-genome sequencing and core genome SNP-based phylogenetic analysis of the Thai samples revealed the dominance of international clone II (Pasteur ST2), which was widely distributed across geographic regions. Carbapenem resistance was primarily mediated by *bla*_OXA-23_, together with intrinsic *bla*_OXA-66_ and resistance-associated mutations in a penicillin-binding protein gene. In addition to OXA-type carbapenemase genes, *bla*_NDM-5_ and *bla*_NDM-1_ were detected in a subset of isolates. In particular, *bla*_NDM-5_ was located within a highly conserved MGE associated with a class 1 integron, IS91 family transposase and IS*Aba125* and was detected across multiple ST2 sublineages. This MGE was distributed across multiple geographic regions and phylogenetic lineages in Thailand, with evidence suggesting widespread horizontal dissemination followed by local clonal expansion. By contrast, *bla*_NDM-1_ was detected less frequently but was consistently associated with a canonical Tn*125* transposon in both ST2 and ST16 isolates, highlighting its continued epidemiological relevance. Collectively, our findings demonstrate that CRAB bloodstream infections in Thailand are driven by a combination of endemic high-risk lineages and the sustained circulation of *bla*_NDM_-harboring MGEs. Overall, our results confirm the importance of continued genomic surveillance for monitoring the emergence and dissemination of *bla*_NDM_-producing CRAB and for strengthening infection prevention and antimicrobial resistance control efforts in hospital settings.

## Introduction

*Acinetobacter baumannii* has emerged as a globally important nosocomial pathogen, causing severe infections, particularly in intensive care units (ICUs) and among immunocompromised patients. This organism is a leading cause of hospital-acquired infections, including ventilator-associated pneumonia and infections of the bloodstream, wounds, urinary tract, and central nervous system [1,2]. Treatment of these infections is now severely limited due to the global spread of antibiotic-resistant *A. baumannii*, particularly carbapenem-resistant *A. baumannii* (CRAB), which is a major contributor to infection-related morbidity, mortality, and healthcare costs [3].

Resistance of *A. baumannii* to carbapenem is most commonly mediated by class D OXA-type carbapenemases, especially *bla*_OXA-23-like_ [4]. Intrinsic *bla*_OXA-51-like_ variants may also contribute to carbapenem resistance, particularly when overexpressed through the presence of upstream insertion sequences such as IS*Aba1* that provide strong promoter activity [5,6]. Additional resistance mechanisms, such as alterations in penicillin-binding proteins or efflux systems, also play a role in carbapenem resistance [7,8]. In recent years, metallo-β-lactamases (MBLs), particularly New Delhi metallo-β-lactamases (NDMs), have emerged as an additional and concerning mechanism of carbapenem resistance in *A. baumannii* [9]. Although *bla*_NDM_ genes are more frequently associated with Enterobacterales, an increasing number of studies are now reporting the presence of *bla*_NDM-1_ and *bla*_NDM-5_ in *A. baumannii*, highlighting an expansion of the host range of these resistance determinants [10,11]. The mobility of *bla*_NDM_ genes is often associated with transposons, insertion sequences, and class 1 integrons, which facilitate the rapid dissemination of these genes across bacterial species and geographic regions [11–15]. This expansion of antibiotic resistance genes has increased the need for global genomic surveillance of CRAB strains.

Whole-genome sequencing (WGS) has become widely used for surveillance of CRAB because it enables high-resolution analysis of population structure, transmission dynamics, and antimicrobial resistance mechanisms [4,11]. When applied to *A. baumannii*, sequence-based typing schemes, such as multilocus sequence typing (MLST), capsular polysaccharide (K-locus) typing, and lipooligosaccharide outer core (O-locus) typing, have provided frameworks for understanding the clonal dissemination and genetic diversity of this species [4,16]. Furthermore, core genome single-nucleotide polymorphism (SNP)–based phylogenetic analysis has facilitated detailed investigations of population structure and genetic relatedness [17]. Integration of genomic data, antimicrobial susceptibility profiles, and epidemiological metadata enables tracking of the emergence and spread of high-risk CRAB lineages and resistance elements [4,16,17].

In Thailand, surveillance data from the National Antimicrobial Resistance Surveillance Center, Thailand (NARST), have documented an increasing burden of *A. baumannii* complex infections, which rose from 8% of all reported bacterial infections in 2000 to 14% in 2022. In parallel, the prevalence of CRAB in Thailand has increased substantially, with 2024 surveillance data indicating resistance rates exceeding 75% overall and over 80% among isolates from ICU settings [18,19]. National surveillance data from 2022 indicated that CRAB was the leading cause of antimicrobial-resistant hospital-origin bloodstream infections. These infections accounted for 51.2% of cases and were associated with mortality rates of up to 60% [20]. While previous studies have described the prevalence of dominant clonal lineages and OXA-type carbapenemases [19–21], comprehensive nationwide genomic data on CRAB bloodstream infections remain limited, particularly with respect to the distribution and genetic context of *bla*_NDM_-harboring elements.

In this study, we performed a genomic surveillance study of CRAB isolates collected from bloodstream infections across Thailand between 2020 and 2024 through NARST, Thai National Institute of Health (Thai NIH), Department of Medical Science (MoPH). Our aim was to define population structure and geographic distribution, characterize antimicrobial resistance gene content, and investigate the dissemination of *bla*_NDM_-harboring MGEs in the Thai CRAB population. Thailand represents a setting with sustained endemic CRAB transmission and high patient mobility, underscoring the need for national-scale genomic surveillance to address this ongoing public health problem. The availability of isolates from multiple regions further enables assessment of regional variation and dissemination patterns with potential relevance to broader regional and global CRAB spread.

## Materials and methods

### CRAB isolates and antimicrobial susceptibility testing

A total of 55 CRAB isolates collected between 2020 and 2024 were included in this study as part of nationwide genomic surveillance conducted through NARST. Isolates were initially identified as *A. baumannii* by participating hospitals using routine clinical microbiology laboratory procedures before submission to NARST. This collection comprised 41 isolates recovered from bloodstream infections and 14 isolates obtained from other clinical sources for comparison. For the purposes of this study, CRAB isolates were accessed from the culture collection between 25/02/2025 and 06/04/2025. All isolates were retrieved from glycerol stocks that had been collected as part of routine antimicrobial resistance surveillance and stored at −80°C until analysis. Prior to biochemical identification, isolates were subcultured on sheep blood agar and incubated at 37°C, overnight. Species identification was reconfirmed using standard biochemical methods according to the Manual of Clinical Microbiology [21].

Antimicrobial susceptibility testing was carried out using the broth microdilution method with customized THAN2F Sensititre plates (Thermo Fisher Scientific, USA). The antimicrobial panel included penicillins and β-lactam/β-lactamase inhibitor combinations (ampicillin, amoxicillin–clavulanate, ampicillin–sulbactam, piperacillin–tazobactam), cephalosporins (cefuroxime, cefoxitin, cefotaxime, ceftriaxone, ceftazidime, cefepime), carbapenems (imipenem, meropenem, doripenem, ertapenem), aminoglycosides (amikacin, gentamicin, netilmicin), fluoroquinolones (ciprofloxacin, levofloxacin), polymyxins (colistin), and folate pathway inhibitors (trimethoprim–sulfamethoxazole). Bacterial inocula were prepared from isolated colonies grown on sheep blood agar, suspended in cation-adjusted Mueller–Hinton broth, and adjusted to a 0.5 McFarland standard using a nephelometer (Thermo Fisher Scientific, USA). A 30 µL aliquot of this suspension was added to 11 mL of Mueller–Hinton broth to prepare the inoculum. Microdilution plates were inoculated using the AIM Sensititre system (Thermo Fisher Scientific, USA), incubated at 35 ± 2°C for 18–24 h, and read with the Sensititre ARIS 2X system (Thermo Fisher Scientific, USA). Colistin susceptibility was determined for all *A. baumannii* isolates using broth microdilution, the reference method recommended by the Clinical and Laboratory Standards Institute (CLSI) [22]. Minimum inhibitory concentrations (MICs) were interpreted in accordance with the CLSI M100, 35^th^ edition, guidelines [22].

### DNA extraction and whole-genome sequencing

CRAB isolates were revived from frozen stocks and grown on sheep blood agar at37°C overnight for genomic DNA extraction. Genomic DNA was extracted and purified using the Presto™ Mini gDNA Bacteria Kit (Geneaid Biotech, Taiwan) following the manufacturer’s instructions. DNA purity and yield were initially assessed using a NanoDrop™ 1000 spectrophotometer (Thermo Scientific, USA), followed by accurate quantification with a Qubit™ 3.0 Fluorometer (Invitrogen, USA). DNA integrity was confirmed using agarose gel electrophoresis. Sequencing libraries were generated from 100 ng of genomic DNA using the Illumina DNA Prep (M) Tagmentation kit (Illumina, USA) and sequenced on an Illumina NextSeq platform with the P1 XLEAP-SBS High Output reagent kit (Illumina, USA), following the manufacturer’s instructions, resulting in 150-bp paired-end reads.

To improve the resolution of comparative genomic analyses, long-read sequencing was performed for seven isolates selected to represent the diversity of *bla*_NDM_ genotypes identified in the collection, including one *bla*_NDM-1_-positive, two *bla*_NDM-5_-positive, and four *bla*_NDM_-negative isolates. Briefly, 50 ng of input DNA was used to prepare barcoded libraries with the Rapid Sequencing gDNA Barcoding kit (SQK-RBK114.96) (Oxford Nanopore Technologies, UK). The libraries were loaded into R10.4.1 flow cells and sequenced on a PromethION P2 Solo system under default operating conditions. Raw signal data were base-called and subjected to quality filtering using Guppy version 6.0.1 [23].

### Genomic assembly and annotation

Raw sequencing reads were initially assessed for quality and trimmed using Trim Galore v0.6.5, followed by genome assembly using SPAdes v4.0.0 via the BV-BRC web resource (https://www.bv-brc.org/) with default parameters [24]. For isolates with long-read sequencing data, *de novo* assembly of combined short-read and long-read sequences was performed using Unicycler v0.4.8 [25]. The assembly quality was evaluated using QUAST v5.0.2 [26]. Taxonomic classification based on average nucleotide identity (ANI) and alignment fraction was performed using GTDB-Tk v2.1.1 [27]. Genome annotation was conducted using Prokka [28]. The genomic regions surrounding carbapenemase genes were examined using the ISfinder database and confirmed by BLAST search to identify associated insertion sequence elements [29]. The Pathogenwatch web tools were used to perform MLST based on the Pasteur and Oxford schemes, as well as K- and O-locus identification [30]. Antimicrobial resistance genes and plasmid replicons were identified using AMRFinderPlus v3.12.8 [31] and the MOB suite [32], respectively.

### Phylogenetic analysis and visualization

Core genome SNPs, including nucleotide substitutions as well as small insertions and deletions, were identified using Snippy v4.6.0. *A. baumannii* strain ACICU (GenBank accession NC_010611.1) was used as the reference genome. Maximum-likelihood phylogenetic analysis was performed using IQ-TREE with the GTR + G nucleotide substitution model and 1,000 bootstrap replicates [33]. Clades with bootstrap support greater than 60% under maximum-likelihood analysis were considered strongly supported. For comparative analysis of the *bla*_NDM-5_ MGE, previously reported *bla*_NDM-5_-positive *A. baumannii* genomes from Thailand [11] were retrieved from public databases and included in a dedicated phylogenetic analysis of *bla*_NDM-5_-positive isolates. The resulting phylogenetic tree was visualized and annotated using the Interactive Tree of Life (iTOL) v7 web tool [34].

### Ethics statement

This study was approved by the Chulabhorn Royal Academy Institutional Review Board (CRA-IRB) on January 16, 2026 (Project Code: IRB004/2569). The study used re-identified clinical isolates collected as part of routine national antimicrobial resistance surveillance, and no patient-identifying information was included. Informed consent was waived by the CRA-IRB. All methods were performed in accordance with the relevant standard guidelines and regulations.

## Results

### Study population and antimicrobial susceptibility profiles

**Fig 1:**
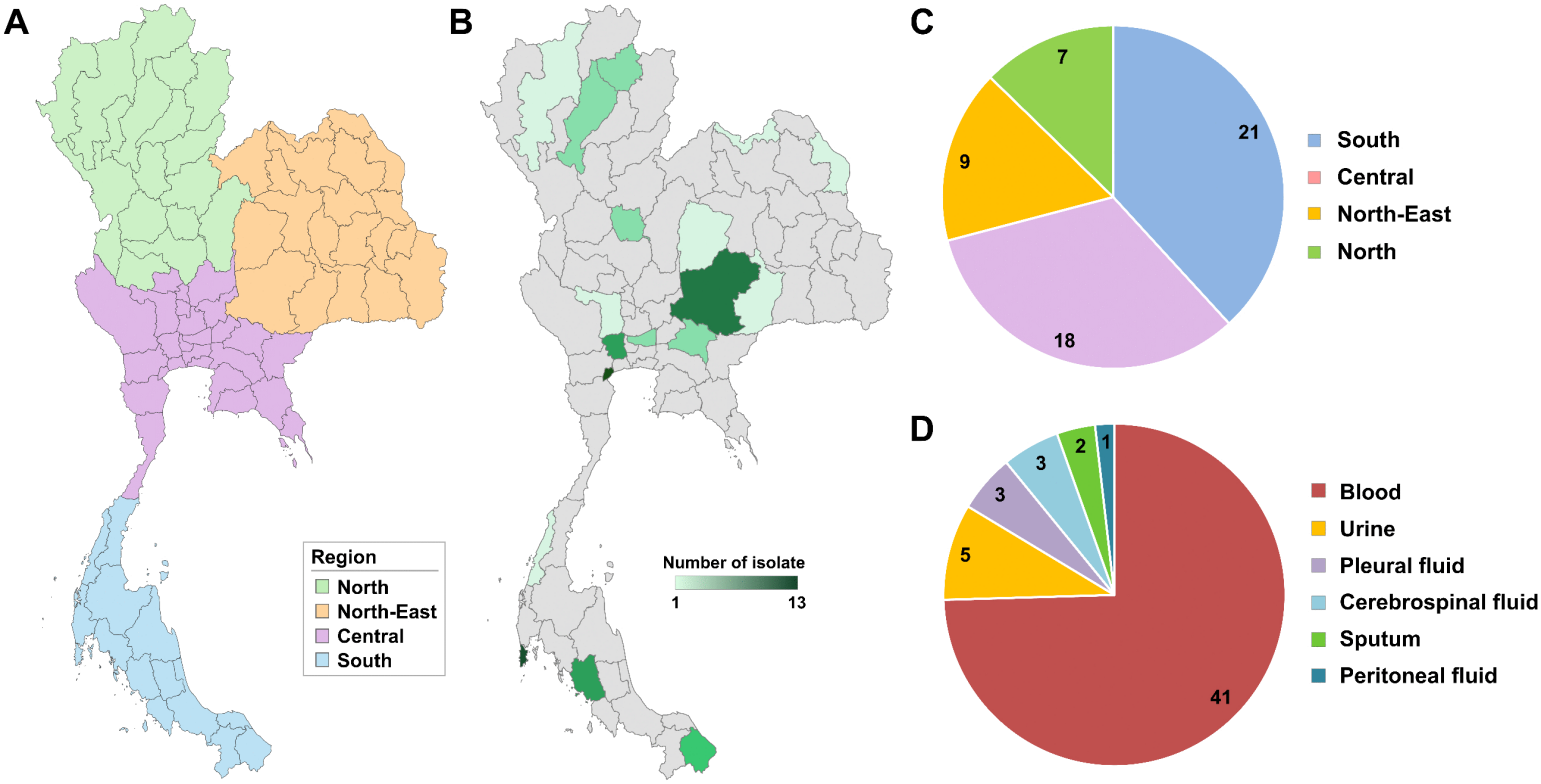
Geographic distribution and specimen sources of CRAB isolates included in this study. (A) Map of Thailand showing the four geographic regions (North, North-East, Central, and South), based on the National Statistical Office of Thailand. (B) Provincial distribution of CRAB isolates, with shading intensity indicating the number of isolates per province. (C) Distribution of isolates by geographic region. (D) Distribution of isolates according to clinical specimen type. Maps were generated by the authors using administrative boundary data obtained from the geoBoundaries database [35]. The geoBoundaries dataset is distributed under the Creative Commons Attribution 4.0 International (CC BY 4.0) license.

A total of 55 clinical isolates of CRAB collected across Thailand during 2020 and 2024 were obtained from the Thai NIH. These isolates originated from multiple hospitals that participate in the NARST network and are located in different geographic regions of Thailand (Fig 1A–C; S1 Table). This study focused primarily on *A. baumannii* bloodstream isolates (n = 41), which accounted for 6.26% (41/655) of all antibiotic-resistant *A. baumannii* isolates from the NARST collection. Isolates from other infection sources, including urine (n = 5), pleural fluid (n = 3), cerebrospinal fluid (n = 3), sputum (n = 2), and peritoneal fluid (n = 1), were included for comparative purposes (Fig 1D; S1 Table).

The antimicrobial susceptibility profiles of 11 antibiotics, representing six different antimicrobial classes (carbapenems, cephalosporins, aminoglycosides, fluoroquinolones, polymyxins, and folate pathway inhibitors) are summarized in S1 Table. All isolates were resistant to imipenem, meropenem, and doripenem. Ertapenem was included in the Sensititre antimicrobial panel; however, the CLSI M100, 35^th^ edition, does not provide ertapenem interpretive breakpoints for *A. baumannii*. Therefore, ertapenem MICs were not interpreted and were not considered in the assessment of carbapenem resistance.

In addition to displaying carbapenem resistance, the majority of isolates (>50 isolates) also exhibited resistance to cephalosporins, aminoglycosides, and fluoroquinolones. Resistance to co-trimoxazole was observed in 43 isolates, whereas colistin resistance was detected in 11 isolates. Notably, eight isolates (14.5%) exhibited resistance to all tested antibiotics and were identified in the North-East, Central, and Southern regions. Additionally, 30 isolates (54.5%) were resistant to all tested antibiotics except colistin and were detected across all regions, indicating that multidrug-resistant *A. baumannii* is endemic in Thailand.

### General genomic features and species confirmation

Complete genome assemblies were generated for seven isolates (AB0001–AB0007) using combined Illumina and Oxford Nanopore Technologies sequencing. The isolates were selected to represent the diversity of *bla*_NDM_ genotypes identified in the collection, including one *bla*_NDM-1_-positive isolate, two *bla*_NDM-5_-positive isolates, and four isolates lacking *bla*_NDM_ genes. The remaining isolates were sequenced using Illumina and assembled as drafts. The assembly quality metrics are summarized in S2 Table. The genome sizes ranged from approximately 3.7 to 4.2 Mb and showed consistent GC content (38.7%–39.5%). Taxonomic analysis confirmed that all isolates belonged to *A. baumannii*, with ANI values ranging from 97.7% to 98.2% relative to the reference genome (S2 Table).

### SNP-based phylogenomic analysis and antimicrobial resistance gene profiling

**Fig 2:**
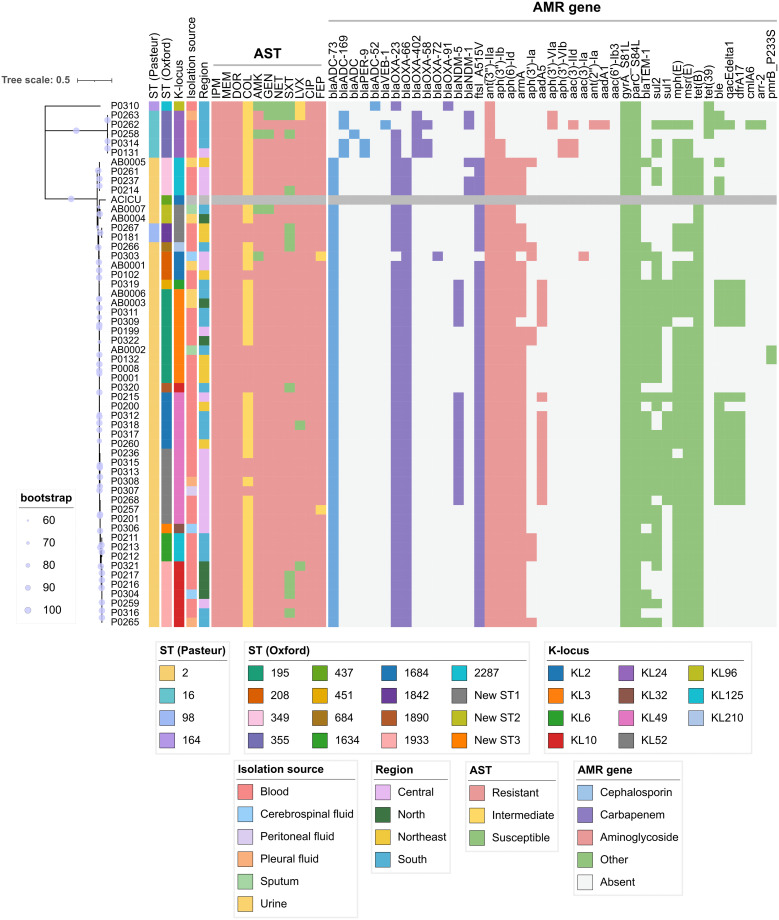
Core genome SNP phylogeny and antimicrobial resistance profiles of CRAB included in this study. A maximum-likelihood phylogenetic tree based on core genome SNPs from 55 CRAB isolates is shown. Annotations include Pasteur and Oxford MLST, K-locus, clinical specimen source, geographic region, antimicrobial susceptibility testing (AST) results, and antimicrobial resistance gene profiles. AST results are shown for imipenem (IPM), meropenem (MEM), doripenem (DOR), amikacin (AMK), gentamicin (GEN), netilmicin (NET), trimethoprim–sulfamethoxazole (SXT), levofloxacin (LVX), ciprofloxacin (CIP), cefepime (FEP), and colistin (COL) and are categorized as resistant (R), intermediate (I), or susceptible (S).

A core genome SNP–based phylogenomic analysis revealed a population structure dominated by a single major lineage corresponding to Pasteur ST2, which comprised the majority of isolates (n = 47; 85.5%) (Fig 2). Although classified as a distinct Pasteur STs, the ST98 isolates (n = 2) clustered within the ST2 clade and shared highly similar antimicrobial resistance gene profiles. Isolates belonging to ST16 (n = 5) and ST164 (n = 1) formed small, distinct clades that were phylogenetically separated from the dominant Pasteur ST2 lineage. All ST16 and ST164 isolates were consistently assigned to Oxford ST355 and Oxford ST2287, respectively, and showed uniform association with specific K-loci (KL34 for ST16 and KL96 for ST164). Isolates belonging to Pasteur ST2 were distributed across multiple Oxford STs, including three newly assigned Oxford STs, indicating greater diversity at the Oxford MLST level. Notably, the K-locus assignments showed complete concordance with the Oxford STs, with all isolates sharing the same Oxford ST carrying the same K-locus. The O-locus distributions were conserved, with Pasteur ST2 isolates predominantly associated with OCL1, whereas the ST164 isolates carried OCL5, and the ST16 isolates were associated with OCL2 and OCL7. Detailed MLST allelic profiles, together with K- and O-locus assignments, are provided in S3 Table. Overall, the phylogenetic clustering was highly consistent with MLST classification using both Pasteur and Oxford schemes as well as with K-locus and O-locus assignments, highlighting the robustness of these approaches for describing the population structure in this dataset.

The dominant ST2 clade was widely distributed across regions and specimen types, whereas the ST16 and ST164 lineages were detected mainly in the Southern region and were largely associated with bloodstream isolates (Fig 2). At the Oxford MLST level, most sequence types showed wide regional and specimen distributions. Notably, Oxford ST1634, a sublineage within Pasteur ST2, was observed exclusively among bloodstream isolates from the Southern region. In addition, a novel Oxford sequence type (New ST1), representing a single-locus variant (SLV) of ST1684 and belonging to the Pasteur ST2 lineage, was detected only in the Central region.

The antimicrobial resistance gene profiling demonstrated a high degree of consistency between phylogenetic clustering and resistance gene content (Fig 2). Carbapenem resistance was primarily associated with the presence of OXA-type carbapenemase genes, particularly *bla*_OXA-23_, which was detected in 46 of the 47 ST2 isolates. The remaining ST2 isolate (P0303) carried *bla*_OXA-72_ instead of *bla*_OXA-23_. In addition to acquired carbapenemase genes, all ST2 isolates harbored the intrinsic *bla*_OXA-66_ gene. Furthermore, all ST2 isolates, except P0303, carried an A515V substitution in the *ftsI* gene that encodes penicillin-binding protein 3 (PBP3). For ST16, all isolates harbored *bla*_OXA-402_ (OXA-51-like beta-lactamase), with some isolates additionally co-harboring *bla*_OXA-23_ and/or *bla*_OXA-58_. No IS*Aba1* or IS*Aba125* elements were detected upstream of OXA-51-like beta-lactamase in any ST16 isolate. The ST164 isolate carried *bla*_OXA-23_ and *bla*_OXA-91_. Notably, no *ftsI* gene mutations were detected in ST16 or ST164 isolates.

In addition to the OXA-type carbapenemase genes, *bla*_NDM-5_ was detected in 17 ST2 isolates distributed across two distinct phylogenetic groups and multiple regions of Thailand. Notably, *bla*_NDM-5_–positive isolates were uniquely associated with other resistant genes, including *aadA5*, *sul1*, *ble*_MBL_, *dfrA17*, and *qacEΔ1* (Fig 2). The *bla*_NDM-1_ gene was also identified in two ST16 isolates from the Southern region and three ST2 isolates, including one from the North-East and two from the Central region, indicating the presence of NDM-type carbapenemases across multiple genetic backgrounds and geographic regions.

All isolates within the ST2 lineage carried the conserved cephalosporin resistance gene *bla*_ADC-73_, whereas ST16 and ST164 isolates harbored *bla*_ADC-169_ and *bla*_ADC-56_, respectively. For aminoglycoside resistance, all isolates possessed the conserved *ant(3ʺ)-IIa* gene. ST2 isolates additionally carried a conserved set of aminoglycoside resistance genes, including *aph(3ʺ)-Ib*, *aph(6)-Id*, and *armA*. In contrast, ST16 isolates showed greater heterogeneity in aminoglycoside resistance gene content, consistent with variability in aminoglycoside susceptibility phenotypes. The ST164 isolate carried only *ant(3ʺ)-IIa* and remained susceptible to all tested aminoglycosides, consistent with the limited substrate spectrum of ANT(3ʺ)-IIa. With respect to colistin resistance, only two ST2 isolates carried a P233S substitution in the *pmrB* gene. For the remaining isolates, no clear association was observed between colistin-resistant phenotypes and known resistance determinants.

Plasmid prediction analysis using MOB-suite identified putative plasmid-associated contigs in several isolates (S4 Table); however, due to fragmented assemblies and limited replicon detection, plasmid localization of resistance genes could not be conclusively resolved.

### Characterization of the *bla*_NDM-5_ MGE

**Fig 3:**
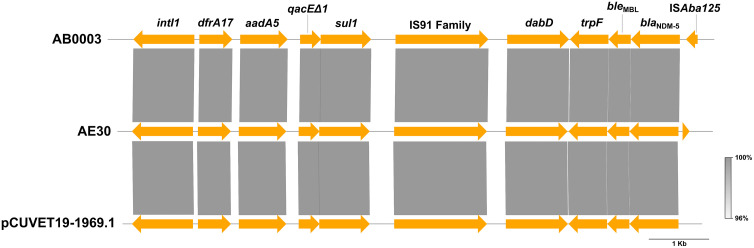
Comparative analysis of the *bla*_NDM-5_ MGE identified in this study. Linear comparison of the *bla*_NDM-5_ MGE from isolate AB0003 (this study), a representative *A. baumannii* isolate from a previous study (AE30; GCA_025214915.1), and an *E. coli* plasmid (pCUVET19-1969.1; NZ_CP098887.1). Open arrows indicate predicted open reading frames and their transcriptional orientations. Grey shaded blocks represent regions of homology, with percentage identity based on BLASTP comparisons.

Further characterization of the *bla*_NDM-5_ MGE identified a class 1 integron containing *intI1*, *dfrA17*, *aadA5*, *qacEΔ1*, and *sul1*, followed by an IS91 family transposase, *dabD*, *trpF*, *ble*_MBL_, *bla*_NDM-5_, and IS*Aba125*. This genetic arrangement has previously been described in *A. baumannii* isolates in Thailand [11]. Fig 3 compares the *bla*_NDM-5_–harboring region of isolate AB0003, a representative isolate from this study, with that of a representative isolate from a previous study (AE30; GCA_025214915.1) [11], showing conserved gene content and organization. Interestingly, further analysis demonstrated that an identical *bla*_NDM-5_ MGE has also been reported on the plasmids of *Escherichia coli* in multiple independent studies. Fig 3 also shows a comparison with a representative *E. coli* plasmid (pCUVET19–1969.1; NZ_CP098887.1), isolated from Thailand [36], revealing an identical *bla*_NDM-5_–associated region. Analysis of the isolate with a complete genome further suggested that the *bla*_NDM-5_ MGE identified in this study is chromosomally encoded rather than plasmid-borne. Notably, isolate P0200 carried a truncated *bla*_NDM-5_ MGE spanning from an IS91 family element to IS*Aba125* and lacked the upstream region from *intI1* to *sul1*.

**Fig 4:**
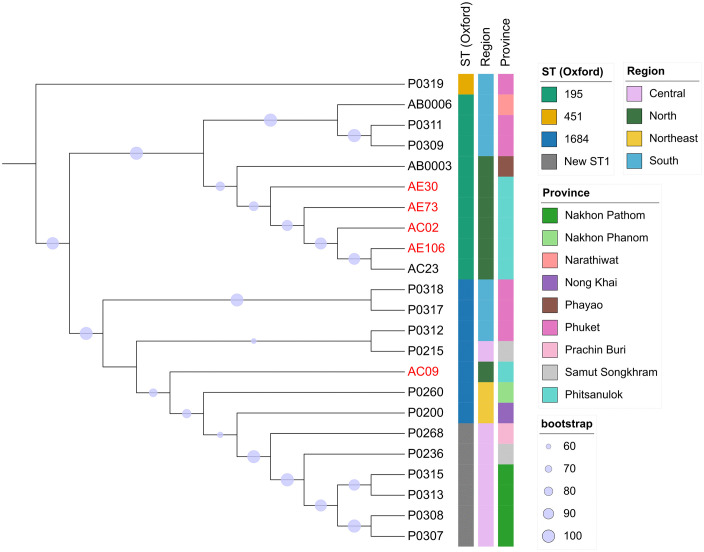
Core genome SNP phylogeny of *A. baumannii* isolates with the *bla*_NDM-5_ MGE in Thailand. A maximum-likelihood phylogenetic tree based on core genome SNPs is shown for *A. baumannii* isolates carrying the *bla*_NDM-5_ MGE, including isolates from the current study and from a previous study [11]. The tree is displayed without branch length scaling to emphasize topological relationships among isolates. Isolates from the previous study are highlighted in red.

As indicated in Fig 2, isolates carrying the *bla*_NDM-5_ MGE in this study did not form a single clade but were instead distributed across different phylogenetic backgrounds. The evolutionary relationships and dissemination of this MGE in Thailand were further investigated by including isolates carrying an identical MGE from a previous study [11] in an expanded core genome SNP–based phylogenetic analysis (Fig 4). Isolates carrying the MGE clustered into three distinct phylogenetic groups. The first group consisted exclusively of Oxford ST195 isolates, which formed two internal subclades: one was composed of isolates from the Southern region and the other comprised isolates from the Northern region, including isolates from the previous study and two isolates from the current study. The second group comprised isolates belonging to Oxford ST1684 and New ST1, an SLV of ST1684. Isolates in this group originated from multiple geographic regions, with one isolate from the previous study (AC09) clustering within this group and representing the only Northern-region isolate in this lineage. In both major groups, several subclades contained isolates from the same province or region, suggesting local clonal expansion. Notably, isolate P0319, the sole representative of Oxford ST451, did not cluster with either of the two major *bla*_NDM-5_–positive groups, indicating a distinct phylogenetic background for this isolate.

### Characterization of the *bla*_NDM-1_–harboring Tn*125* transposon

**Fig 5:**
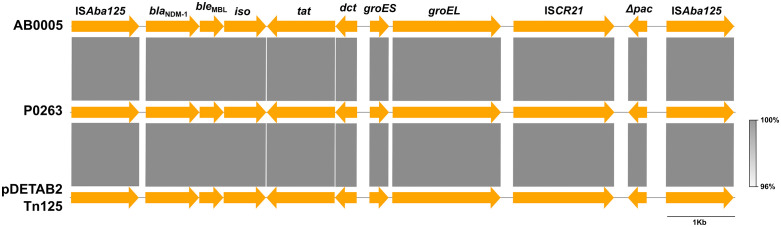
Genetic organization of the *bla*_NDM-1_–harboring Tn*125* transposon in *A. baumannii* isolates. Schematic comparison of the *bla*_NDM-1_–harboring Tn*125* region from isolate AB0005 (Pasteur ST2), isolate P0263 (Pasteur ST16), and the Tn*125* transposon from plasmid pDETAB2 (CP047975.1) reported in a previous study. Gray shaded blocks indicate regions of high sequence similarity based on protein BLASTP comparisons.

This study identified five *bla*_NDM-1_–positive isolates, including three ST2 isolates and two ST16 isolates. Despite their phylogenetic divergence, all *bla*_NDM-1_–positive isolates carried an identical canonical Tn*125* transposon associated with *bla*_NDM-1_. This Tn*125* transposon was flanked by IS*Aba125* and contained genes typically associated with Tn*125*, including *groES*, *groEL*, *bla*_MBL_ and IS*CR21*. The same Tn*125* structure has been reported for plasmids of *A. baumannii* [37]. Fig 5 compares the *bla*_NDM-1_–harboring region from isolate AB0005 (a representative ST2 isolate) and isolate P0263 (a representative ST16 isolate) with the Tn*125* region of plasmid pDETAB2 (GenBank accession: CP047975.1) described in a previous study [37], demonstrating conserved gene content and organization. Because the genome of AB0005 was fully resolved, further analysis confirmed that Tn*125* is chromosomally located in this isolate. For the remaining *bla*_NDM-1_–positive isolates, the availability only of short-read genome assemblies precluded definitive assignment of the Tn*125* element to a chromosomal or plasmid location.

## Discussion

In this nationwide genomic surveillance study, we investigated the population structure and antimicrobial resistance profiles of CRAB isolates from bloodstream infections across Thailand between 2020 and 2024, with particular focus on the dissemination of *bla*_NDM_-associated MGEs. The CRAB population in this study was dominated by Pasteur ST2, belonging to the international clone II (IC2). This finding agrees with the data from multiple previous surveillance studies from Thailand, which have also reported ST2 as the predominant lineage among clinical CRAB isolates and its frequent association with multidrug resistance and hospital outbreaks [11,38–40]. Our data extend these observations by demonstrating that ST2 is not only widespread geographically but also represents the predominant lineage potentially responsible for bloodstream infections nationwide. Other STs found in our samples, including ST16, ST98, and ST164, have also been reported previously in Thailand [38–40]. Although ST98 was assigned as a distinct Pasteur ST, our SNP-based phylogenomic analysis showed that ST98 isolates clustered within the ST2 clade and shared highly similar resistance gene profiles. This supports prior observations that minor ST variants may represent sublineages within IC2 [41].

Phylogenetic clustering in this study showed a high degree of concordance with both the Pasteur and Oxford MLST schemes, as well as with K- and O-locus assignments, supporting the robustness of these typing frameworks for describing CRAB population structure. The strong association observed between Oxford STs and specific K-loci is consistent with previous reports [17] and likely reflects limited recombination at the K-locus within defined clonal backgrounds [17]. This linkage provides additional evidence for the use of capsular typing as a marker of clonal relatedness in *A. baumannii* [42]. Notably, the Oxford MLST scheme enabled fine-scale discrimination of closely related sublineages within the dominant ST2 lineage, particularly those carrying *bla*_NDM-5_. This higher resolution greatly improves genomic surveillance as it facilitates more precise tracking of clonal expansion and outbreak dynamics within established investigation criteria for high-risk lineages in a hospital setting. A key finding is that Oxford MLST can resolve diversity that is not distinguished under the Pasteur scheme, as this capacity could prove useful for short-term epidemiological investigations and local outbreak detection [17,43]. However, despite this increased discriminatory power, the Oxford MLST scheme has known limitations, most notably the presence of paralogous copies of the *gdhB* gene, which can lead to ambiguous allele calling and the generation of artefactual STs [44]. These issues highlight the importance of interpreting Oxford MLST results alongside core genome phylogenetic analysis and additional genomic markers to ensure robust lineage assignment.

As expected, carbapenem resistance in this collection was primarily driven by OXA-type carbapenemases. The near-universal presence of *bla*_OXA-23_ among our ST2 isolates was consistent with findings from earlier surveillance studies in Thailand and reflects the long-standing establishment of this resistance determinant in the region [38–40]. The consistent detection of the intrinsic *bla*_OXA-66_ gene and A515V substitution in *ftsI* (PBP3) among ST2 isolates further highlights the multilayered mechanisms of carbapenem resistance in this lineage [45–47]. Although occurring less frequently than OXA-type carbapenemases, *bla*_NDM-5_ was detected in multiple ST2 isolates distributed across distinct phylogenetic groups and geographic regions. This pattern indicates repeated acquisition events rather than expansion of a single clonal lineage [48]. Detailed analysis revealed that *bla*_NDM-5_ was embedded within a highly conserved MGE, with additional resistance genes including *dfrA17* (trimethoprim resistance), *aadA5* (aminoglycoside resistance), *sul1* (sulfonamide resistance), *ble*_MBL_ (bleomycin resistance), and *qacEΔ1* (disinfectant tolerance). This *bla*_NDM-5_ MGE has been previously reported in *A. baumannii* isolates from clinical specimens and hospital environments in Thailand collected between 2020 and 2021 [11]. Our findings demonstrate that the same *bla*_NDM-5_ MGE has persisted in Thailand over multiple years. This temporal continuity, combined with the identical genetic structures observed across studies and regions, indicates ongoing circulation of this MGE within the national CRAB population and suggests the potential for cross-border dissemination. Although all isolates carrying the *bla*_NDM-5_ MGE from both the current and previous studies belonged to Pasteur ST2, they segregated into multiple Oxford STs, indicating sublineage diversification within this genetic background.

The SNP-based phylogenetic analysis of isolates carrying the *bla*_NDM-5_ MGE provides important insights into the dissemination dynamics of this resistance element within the Thai CRAB population. Rather than clustering into a single monophyletic lineage, *bla*_NDM-5_-positive isolates were distributed across multiple phylogenetic subgroups, indicating that the spread of this *bla*_NDM-5_ MGE is not driven solely by clonal expansion of a single successful strain. This pattern strongly supports a model of horizontal transmission of the MGE across distinct genetic backgrounds, followed by local clonal amplification within specific lineages [49]. The separation of *bla*_NDM-5_ carrying isolates into three phylogenetic groups further highlights the complexity of its dissemination. The largest group, composed exclusively of Oxford ST195 isolates, showed clear subclade structure corresponding to geographic origin, with distinct clusters associated with Northern and Southern regions. This regional clustering suggests sustained local transmission following acquisition of the *bla*_NDM-5_ MGE. Notably, Oxford ST195 isolates carrying this MGE from the previous study were recovered from hospital environments, supporting the possibility of persistent environmental reservoirs contributing to ongoing transmission within healthcare settings [11]. In contrast, the group comprising Oxford ST1684 and its SLV (New ST1) included isolates from multiple regions, suggesting broader dissemination across Thailand, potentially facilitated by patient movement or inter-hospital transfer [50,51]. Additionally, the placement of isolate P0319 (Oxford ST451) outside the two dominant *bla*_NDM-5_ groups underscores that acquisition of this *bla*_NDM-5_ MGE can also occur sporadically in unrelated genetic backgrounds, further emphasizing its mobility.

Importantly, the same *bla*_NDM-5_ MGE has been reported in plasmids of *E. coli* in multiple studies from Asian countries, including China, Myanmar, and Thailand, and has been identified in isolates from different sources, including clinical, animal, and environmental sources [36,52–55]. In Thailand, *E. coli* carrying plasmids with an identical *bla*_NDM-5_ MGE have been reported from clinical isolates collected during 2015–2016 [54], as well as domestic animal infections, including cats and dogs [36]. The presence of an identical MGE across diverse hosts and ecological niches suggests that, in addition to intra-species gene transfer within *A. baumannii*, *E. coli* may serve as an important reservoir and vehicle for inter-species dissemination of this resistance element. Its detection in both *Enterobacterales* and non-fermenting Gram-negative bacteria such as *A. baumannii* underscores the ability of MGEs to cross taxonomic boundaries [56].

The *bla*_NDM-1_ gene was detected in both ST2 and ST16 isolates and was consistently associated with a canonical Tn*125* transposon flanked by IS*Aba125*. This *bla*_NDM-1_–harboring Tn*125* has been reported on both the chromosome and plasmids of *A. baumannii* [37,57]. The conserved gene content and synteny of this Tn*125* across phylogenetically distinct isolates indicate dissemination through independent transfer of a MGE rather than clonal expansion. The chromosomal localization of this Tn*125* in at least one fully resolved genome suggests stabilization of this element within certain CRAB lineages, as has been described previously [58,59]. Although *bla*_NDM-1_ was detected at a lower frequency than *bla*_NDM-5_, previous reports have shown that NDM-1–producing CRAB has been implicated in both hospital-associated outbreaks and regional dissemination [14,15,58,60]. These observations indicate that, despite its lower prevalence in the present collection, *bla*_NDM-1_ remains an epidemiologically important resistance determinant and should continue to be monitored through national genomic surveillance efforts.

Collectively, our findings highlight the complementary roles of clonal expansion and horizontal gene transfer in shaping the CRAB population in Thailand. While IC2 remains the dominant lineage driving bloodstream infections, the parallel circulation of MGE enables resistance determinants to persist and spread independently of clonal background. This dual mechanism promotes both the long-term stability of high-risk clones and the continual emergence of new resistant variants, complicating infection prevention and antimicrobial stewardship efforts. These findings underscore the importance of established national genomic surveillance frameworks for simultaneously tracking population structure and resistance gene mobility, particularly among antimicrobial-resistant bacteria prioritized by the World Health Organization (WHO) [61].

While this study provides detailed insights into the genomic epidemiology of CRAB in Thailand, additional studies are required to better characterize the genomic diversity and transmission dynamics of CRAB in Thailand. Although isolates were collected through a nationwide surveillance network and represented multiple geographic regions of Thailand, the relatively limited number of bloodstream CRAB isolates available for genomic analysis may not fully capture the genetic diversity and resistance mechanisms circulating among CRAB populations nationwide. In addition, the study focused exclusively on bloodstream isolates and therefore may not reflect the genomic characteristics of CRAB circulating in other clinical sources or environmental reservoirs. Plasmid reconstruction was limited by the predominant use of short-read sequencing for most isolates. Moreover, integration of detailed clinical outcome data will be essential to more effectively link genomic features with disease severity, treatment response, and patient outcomes. Future studies incorporating long-read sequencing, broader sampling strategies, and clinical metadata will be critical for comprehensively resolving population diversity and the transmission dynamics of mobile resistance elements and informing their prevention and control in clinical settings among CRAB in Thailand.

## Conclusions

This nationwide genomic surveillance study provides a comprehensive overview of the population structure and resistance mechanisms of CRAB causing bloodstream infections in Thailand. The CRAB population was dominated by the IC2 lineage (Pasteur ST2), which was widely distributed across geographic regions, underscoring its continued circulation and clinical importance. Beyond clonal dissemination, our findings highlight the critical role of MGEs in shaping resistance diversity within CRAB. Both *bla*_NDM-5_ and *bla*_NDM-1_ were identified across multiple genetic backgrounds and were carried within conserved MGEs that enable horizontal dissemination. The widespread distribution of a *bla*_NDM-5_ MGE across ST2 sublineages from multiple regions indicates its sustained circulation throughout Thailand, suggesting the establishment of high-risk resistance elements within endemic CRAB lineages and posing an ongoing challenge to infection control efforts. Continued genomic surveillance incorporating high-resolution phylogenetics and detailed analysis of resistance gene contexts will be essential to monitor the emergence, persistence, and spread of NDM-producing CRAB and to inform effective infection prevention and AMR control strategies.

## Supporting information

S1 Table Metadata and antimicrobial susceptibility profiles of carbapenem-resistant *Acinetobacter baumannii* isolates included in this study. (XLSX)

S2 Table Genome assembly statistics and taxonomic assignment of carbapenem-resistant *Acinetobacter baumannii* isolates included in this study. (XLSX)

S3 Table Multilocus sequence typing (MLST), K-locus, and O-locus assignments of carbapenem-resistant *Acinetobacter baumannii* isolates included in this study. (XLSX)

S4 Table Putative plasmid-associated contigs and plasmid prediction results identified by MOB-suite. (XLSX)
