# Supplementary material for: Genomic surveillance of carbapenem-resistant Acinetobacter baumannii from bloodstream infections in Thailand reveals widespread dissemination of blaNDM–harboring mobile genetic elements
Source: PLoS One. 2026 Jul 13;21(7):e0353488. doi: 10.1371/journal.pone.0353488 (PMC13362128; doi:10.1371/journal.pone.0353488)
Supplement: Attachment — Submitted filename: Response to Reviewers.pdf [file pone.0353488.s006.pdf]

Responses to Journal Requirements

1. When submitting your revision, we need you to address these additional requirements.

Please ensure that your manuscript meets PLOS ONE's style requirements, including those for file naming. The PLOS ONE style templates can be found at

https://journals.plos.org/plosone/s/file?id=wjVg/PLOSOne_formatting_sample_main_body.pdf and

https://journals.plos.org/plosone/s/file?id=ba62/PLOSOne_formatting_sample_title_authors_affiliations.pdf

We have reviewed the manuscript and confirmed that it has been formatted according to the PLOS ONE style requirements and file naming guidelines. The revised submission complies with the formatting templates provided by the journal.

2. Please note that your Data Availability Statement is currently missing the direct link to access each database. If your manuscript is accepted for publication, you will be asked to provide these details on a very short timeline. We therefore suggest that you provide this information now, though we will not hold up the peer review process if you are unable.

We have revised the Data Availability Statement to include the direct link to the BioProject database where the whole-genome sequencing data are publicly available.

Changes in manuscript:

The Data Availability Statement has been updated as follows:

" The WGS data obtained from the isolates analyzed in this study are available under BioProject accession number PRJNA1391494 in the NCBI database (https://www.ncbi.nlm.nih.gov/bioproject/PRJNA1391494/)."

3. We note that there is identifying data in the Supporting Information file <S1 Table>. Due to the inclusion of these potentially identifying data, we have removed this file from your file inventory. Prior to sharing human research participant data, authors should consult with an ethics committee to ensure data are shared in accordance with participant consent and all applicable local laws.

Data sharing should never compromise participant privacy. It is therefore not appropriate to publicly share personally identifiable data on human research participants. The following are examples of data that should not be shared:

- Name, initials, physical address
- Ages more specific than whole numbers
- Internet protocol (IP) address
- Specific dates (birth dates, death dates, examination dates, etc.)
- Contact information such as phone number or email address
- Location data
- ID numbers that seem specific (long numbers, include initials, titled “Hospital ID”) rather than random (small numbers in numerical order)

Data that are not directly identifying may also be inappropriate to share, as in combination they can become identifying. For example, data collected from a small group of participants, vulnerable populations, or private groups should not be shared if they involve indirect identifiers (such as sex, ethnicity, location, etc.) that may risk the identification of study participants.

Additional guidance on preparing raw data for publication can be found in our Data Policy (https://journals.plos.org/plosone/s/data-availability#loc-human-research-participant-data-and-other-sensitive-data) and in the following article: http://www.bmj.com/content/340/bmj.c181.long.

Please remove or anonymize all personal information (<specific identifying information in file to be removed>), ensure that the data shared are in accordance with participant consent, and re-upload a fully anonymized data set. Please note that spreadsheet columns with personal information must be removed and not hidden as all hidden columns will appear in the published file.

We carefully reviewed the Supporting Information file (S1 Table) and identified that the province-level geographic information associated with individual clinical isolates could potentially be considered an indirect identifier. To further protect participant privacy and ensure compliance with the PLOS ONE Data Policy, we have removed the Province column from S1 Table and retained only the broader geographic region classification (North, Northeast, Central, and South).

The revised S1 Table does not contain any direct personal identifiers, hospital identifiers, exact collection dates, or other information that could reasonably be used to identify individual participants. The data presented are fully consistent with the approved ethical framework and participant confidentiality requirements.

4. Your ethics statement should only appear in the Methods section of your manuscript. If your ethics statement is written in any section besides the Methods, please move it to the Methods section and delete it from any other section. Please ensure that your ethics statement is included in your manuscript, as the ethics statement entered into the online submission form will not be published alongside your manuscript.

In accordance with PLOS ONE requirements, we have moved the Ethics Statement to the Materials and Methods section of the manuscript and removed the standalone Ethics Statement section from the end of the manuscript.

5. Please upload a new copy of Figure 2, as the detail is not clear. Please follow the link for more information: https://journals.plos.org/plosone/s/figures

We have revised Figure 2 to improve readability by increasing the figure dimensions, enlarging text labels, and exporting the figure at higher resolution in accordance with the PLOS ONE figure guidelines. The revised figure has been uploaded with the resubmission.

6. We note that Figures 1a and 1b in your submission contain map images which may be copyrighted. All PLOS content is published under the Creative Commons Attribution License (CC BY 4.0), which means that the manuscript, images, and Supporting Information files will be freely available online, and any third party is permitted to access, download, copy, distribute, and use these materials in any way, even commercially, with proper attribution. For these reasons, we cannot publish previously copyrighted maps or satellite images created using proprietary data, such as Google software (Google Maps, Street View, and Earth). For more information, see our copyright guidelines: http://journals.plos.org/plosone/s/licenses-and-copyright.

We require you to either present written permission from the copyright holder to publish these figures specifically under the CC BY 4.0 license, or remove the figures from your submission:

a. You may seek permission from the original copyright holder of Figures 1a and 1b to publish the content specifically under the CC BY 4.0 license.

We recommend that you contact the original copyright holder with the Content Permission Form (http://journals.plos.org/plosone/s/file?id=7c09/content-permission-form.pdf) and the following text:

"I request permission for the open-access journal PLOS ONE to publish XXX under the Creative Commons Attribution License (CCAL) CC BY 4.0 (http://creativecommons.org/licenses/by/4.0/). Please be aware that this license allows unrestricted use and distribution, even commercially, by third parties. Please reply and provide explicit written permission to publish XXX under a CC BY license and complete the attached form."

Please upload the completed Content Permission Form or other proof of granted permissions as an "Other" file with your submission.

In the figure caption of the copyrighted figure, please include the following text: "Reprinted from [ref] under a CC BY license, with permission from [name of publisher], original copyright [original copyright year]."

b. If you are unable to obtain permission from the original copyright holder to publish these figures under the CC BY 4.0 license or if the copyright holder's requirements are incompatible with the CC BY 4.0 license, please either i) remove the figure or ii) supply a replacement figure that complies with the CC BY 4.0 license. Please check copyright information on all replacement figures and update the figure caption with source information. If applicable, please specify in the figure caption text when a figure is similar but not identical to the original image and is therefore for illustrative purposes only.

To ensure compliance with the PLOS ONE copyright and licensing requirements, we have removed the original map images and replaced them with newly generated maps created by the authors using administrative boundary data obtained from the geoBoundaries database (https://www.geoboundaries.org/). The geoBoundaries dataset is distributed under the Creative Commons Attribution 4.0 International (CC BY 4.0) license, which is fully compatible with the PLOS ONE publication license. Therefore, no additional permission from a third-party copyright holder is required.

A revised Figure 1 has been generated and uploaded with the resubmission. Figures 1A and 1B have been replaced with newly generated maps based on geoBoundaries administrative boundary data. The figure legend has been revised to acknowledge the geoBoundaries dataset

and its associated publication. In addition, the geoBoundaries reference has been added to the reference list and cited in the figure legend in accordance with the dataset attribution requirements.

The original Figure 1 legend:

"Fig 1. Geographic distribution and specimen sources of CRAB isolates included in this study. (A) Map of Thailand showing the four geographic regions (North, North-East, Central, and South), based on the National Statistical Office of Thailand. (B) Provincial distribution of CRAB isolates, with shading intensity indicating the number of isolates per province. (C) Distribution of isolates by geographic region. (D) Distribution of isolates according to clinical specimen type. Maps were generated by the authors in Microsoft Excel (version 2507 build 16.0.19029.20136) using embedded OpenStreetMap data (© OpenStreetMap contributors, https://www.openstreetmap.org), licensed under the Open Database License (ODbL) by the OpenStreetMap Foundation (OSMF)."

has been revised to:

"Fig 1. Geographic distribution and specimen sources of CRAB isolates included in this study. (A) Map of Thailand showing the four geographic regions (North, North-East, Central, and South), based on the National Statistical Office of Thailand. (B) Provincial distribution of CRAB isolates, with shading intensity indicating the number of isolates per province. (C) Distribution of isolates by geographic region. (D) Distribution of isolates according to clinical specimen type. Maps were generated by the authors using administrative boundary data obtained from the geoBoundaries database [32]. The geoBoundaries dataset is distributed under the Creative Commons Attribution 4.0 International (CC BY 4.0) license."

7. Please include captions for your Supporting Information files at the end of your manuscript, and update any in-text citations to match accordingly. Please see our Supporting Information guidelines for more information: http://journals.plos.org/plosone/s/supporting-information.

We have added a Supporting Information section at the end of the manuscript containing captions for all Supporting Information files. In-text citations have been reviewed and updated where necessary to ensure consistency with the Supporting Information file names and captions.

Responses to Reviewers' comments

Reviewer #1: The study is good and the authors have demonstrated a good grasp of the problem they tried to address. The manuscript is well written and technically sound. The authors should provide details of how the bacteria were subcultured prior to biochemical identification and genomic studies.

We thank the reviewer for the positive evaluation of our study and for this helpful suggestion. Additional details regarding isolate subculturing prior to biochemical identification have been added to the Materials and Methods section.

The original text:

"All isolates were retrieved from glycerol stocks that had been collected as part of routine antimicrobial resistance surveillance and stored at −80°C until analysis. Species identification was reconfirmed using standard biochemical methods according to the Manual of Clinical Microbiology"

Has been revised to:

" All isolates were retrieved from glycerol stocks that had been collected as part of routine antimicrobial resistance surveillance and stored at −80°C until analysis. Prior to biochemical identification, isolates were subcultured on sheep blood agar and incubated at 37°C, overnight. Species identification was reconfirmed using standard biochemical methods according to the Manual of Clinical Microbiology"

Reviewer #2: The study was well researched and findings well presented. Apart from lack of statistical backing, every aspect of the write is impeccably written. The findings made represent the current situation and calls for a continuation of genomic surveillance to monitor the emergence and dissemination of blaNDM-producing CRAB and also to strengthen infection prevention and antimicrobial resistance control efforts in hospital settings. This is a very important call as it represents a critical need in our global health system

We thank the reviewer for the positive evaluation of our study and for recognizing the importance of continued genomic surveillance of carbapenem-resistant *Acinetobacter baumannii*. We appreciate the reviewer's support for our conclusions regarding the need for

ongoing monitoring of *bla*$_{NDM}$-producing CRAB and for strengthened infection prevention and antimicrobial resistance control efforts.

Reviewer #3: Antibiotic resistance is a major health threat and Acinetobacter baumannii is one of the pathogens included in the critical level of the WHO priority list. Unravelling resistance determinants and distribution patterns of resistant isolates is extremely important to tackle bacterial infections due to multidrug resistant species. In this study, the authors explore the resistant genes and associated mobile genetic elements associated with carbapenem-resistant A. baumannii from bloodstream infections in Thailand. The authors have done a nationwide study, collecting isolates from multiple regions. However, only 41 isolates (+14 from other sources) were taken into consideration, which I find a little low to represent one country and see it as the main limitation of the study. Another weakness of the study as it is, is that the authors de not fully explore the information obtained by WGS (for instance, regarding detection of transposition associated with NDM-carrying mobile genetic elements).

We thank the reviewer for the thoughtful evaluation of our study and for recognizing the importance of investigating resistance determinants and mobile genetic elements among carbapenem-resistant *Acinetobacter baumannii*. We acknowledge the limitations associated with the sample size and have highlighted this point in the Discussion section.

The following sentence has been added to the Discussion:

"While this study provides detailed insights into the genomic epidemiology of CRAB in Thailand, additional studies are required to better characterize the genomic diversity and transmission dynamics of CRAB in Thailand. Although isolates were collected through a nationwide surveillance network and represented multiple geographic regions of Thailand, the relatively limited number of bloodstream CRAB isolates available for genomic analysis may not fully capture the genetic diversity and resistance mechanisms circulating among CRAB populations nationwide. In addition, the study focused exclusively on bloodstream isolates and therefore may not reflect the genomic characteristics of CRAB circulating in other clinical sources or environmental reservoirs."

Our study focused primarily on bloodstream isolates collected through the national surveillance network, which limited the number of available isolates but allowed us to investigate CRAB associated with severe invasive infections from multiple regions of Thailand.

We also appreciate the reviewer's comment regarding the utilization of WGS data. In this study, we aimed to focus on the characterization and dissemination of *bla*$_{NDM}$-associated mobile genetic elements. Accordingly, we performed detailed analyses of *bla*$_{NDM-5}$–harboring integrons, *bla*$_{NDM-1}$–associated Tn*125* transposons, their genomic contexts, and their distribution across phylogenetic lineages and geographic regions. Detailed responses to the specific comments are provided below.

Specific comments

Lines 59-61 -OXA-51-like variants is only related to carbapenem resistance when associated with insertion sequences such as ISAba1 or ISAba125, which work as the promotor for the gene expression. This fact is never mentioned during the text, including during the analysis of the results of the genes carried by the studied isolates. WGS was performed and the presence or absence of such insertion sequences was not acknowledged (lines 258-262).

We thank the reviewer for this important comment. We agree that OXA-51-like carbapenemases are most commonly associated with carbapenem resistance when overexpressed through upstream insertion sequences such as IS*Aba1*.

To address this concern, we revised the Introduction to clarify the role of insertion sequence-mediated overexpression of OXA-51-like carbapenemases. In addition, we examined the upstream genomic regions of *bla*$_{OXA-402}$ in all ST16 isolates using the ISfinder database and BLAST searches and found that no ISAba1 or ISAba125 elements were present upstream of the gene. This information has been added to the Results section. We have also updated the Materials and Methods section to describe the approach used to identify insertion sequence elements associated with carbapenemase genes.

Changes in manuscript:

Introduction

The original text:

"Resistance of *A. baumannii* to carbapenem is most commonly mediated by class D OXA-type carbapenemases, especially *bla*$_{OXA-23-like}$, and often in combination with intrinsic *bla*$_{OXA-51-like}$ variants"

has been revised to:

"Resistance of *A. baumannii* to carbapenem is most commonly mediated by class D OXA-type carbapenemases, especially *bla*$_{OXA-23-like}$ [4]. Intrinsic *bla*$_{OXA-51-like}$ variants may also contribute to carbapenem resistance, particularly when overexpressed through the presence of upstream insertion sequences such as IS*Aba1* that provide strong promoter activity [5,6]."

Materials and methods

The following text has been added to: Genomic assembly and annotation

"The genomic regions surrounding carbapenemase genes were examined using the ISfinder database and confirmed by BLAST search to identify associated insertion sequence elements [29]."

Results

The original text:

"For ST16, all isolates harbored *bla*$_{OXA-402}$ (OXA-51-like beta-lactamase), with some isolates additionally co-harboring *bla*$_{OXA-23}$ and/or *bla*$_{OXA-58}$."

has been revised to:

"For ST16, all isolates harbored *bla*$_{OXA-402}$ (OXA-51-like beta-lactamase), with some isolates additionally co-harboring *bla*$_{OXA-23}$ and/or *bla*$_{OXA-58}$. No IS*Aba1* or IS*Aba125* elements were detected upstream of OXA-51-like beta-lactamase in any ST16 isolate."

Lines 114-115 – "Species identification was reconfirmed…". What was the original identification method?

We thank the reviewer for this comment. The isolates were initially identified as A. *baumannii* by participating hospitals as part of routine clinical microbiology laboratory procedures before submission to NARST. Species identification was subsequently reconfirmed prior to genomic analysis. We have clarified this information in the Materials and Methods section.

The original text:

"A total of 55 CRAB isolates collected between 2020 and 2024 were included in this study as part of nationwide genomic surveillance conducted through NARST."

has been revised to:

"A total of 55 CRAB isolates collected between 2020 and 2024 were included in this study as part of nationwide genomic surveillance conducted through NARST. Isolates were initially identified as *A. baumannii* by participating hospitals using routine clinical microbiology laboratory procedures before submission to NARST."

Lines 176-178 – The authors chose to analyse the A. baumannii bloodstream isolates, which only account for 6.26 % of the total A. baumannii isolates they had available. Why choosing this? Nothing is written to justify this choice.

We thank the reviewer for this comment. The rationale for focusing on bloodstream isolates was based on their substantial clinical significance and was described in the Introduction.

Specifically, we stated that:

"National surveillance data from 2022 indicated that CRAB was the leading cause of antimicrobial-resistant hospital-origin bloodstream infections. These infections accounted for 51.2% of cases and were associated with mortality rates of up to 60% [20]. While previous studies have described the prevalence of dominant clonal lineages and OXA-type carbapenemases [19–21], comprehensive nationwide genomic data on CRAB bloodstream infections remain limited, particularly with respect to the distribution and genetic context of bla_{NDM}-harboring elements."

Therefore, we focused our genomic analysis on bloodstream isolates because they represent a severe form of *A. baumannii* infection associated with high mortality, while also addressing an important knowledge gap regarding the nationwide molecular epidemiology and genomic characteristics of CRAB bloodstream infections in Thailand.

Lines 192-194 – Carbapenems, cephalosporins, aminoglycosides, fluoroquinolones are antimicrobial classes, but cotrimoxazole and colistin no.

We thank the reviewer for this comment. We agree that colistin and trimethoprim–sulfamethoxazole are individual antimicrobial agents. To improve consistency, we have revised the text to classify all antimicrobials according to their respective antimicrobial classes.

Materials and Methods

The original text:

"The antimicrobial panel included penicillins and β-lactam/β-lactamase inhibitor combinations (ampicillin, amoxicillin–clavulanate, ampicillin–sulbactam, piperacillin–tazobactam), cephalosporins (cefuroxime, cefoxitin, cefotaxime, ceftriaxone, ceftazidime, cefepime), carbapenems (imipenem, meropenem, doripenem, ertapenem), aminoglycosides (amikacin, gentamicin, netilmicin), fluoroquinolones (ciprofloxacin, levofloxacin), colistin, and trimethoprim–sulfamethoxazole."

has been revised to:

"The antimicrobial panel included penicillins and β-lactam/β-lactamase inhibitor combinations (ampicillin, amoxicillin–clavulanate, ampicillin–sulbactam, piperacillin–tazobactam), cephalosporins (cefuroxime, cefoxitin, cefotaxime, ceftriaxone, ceftazidime, cefepime), carbapenems (imipenem, meropenem, doripenem, ertapenem), aminoglycosides (amikacin, gentamicin, netilmicin), fluoroquinolones (ciprofloxacin, levofloxacin), polymyxins (colistin), and folate pathway inhibitors (trimethoprim–sulfamethoxazole)."

Results

The original text:

"The antimicrobial susceptibility profiles of 11 antibiotics, representing six different antimicrobial classes (carbapenems, cephalosporins, aminoglycosides, fluoroquinolones, co-trimoxazole, and colistin), are summarized in S1 Table."

has been revised to:

"The antimicrobial susceptibility profiles of 11 antibiotics, representing six different antimicrobial classes (carbapenems, cephalosporins, aminoglycosides, fluoroquinolones, polymyxins, and folate pathway inhibitors) are summarized in S1 Table."

Lines 194-195 – "All isolates were resistant to all tested carbapenems, including imipenem, meropenem, and doripenem". What about ertapenem? Either the isolates are resistant to all tested carbepenems, which include ertapenem, or are not resistant to all.

We thank the reviewer for this comment. Although ertapenem was included in the antimicrobial susceptibility testing panel, antimicrobial susceptibility results were interpreted according to the Clinical and Laboratory Standards Institute (CLSI) M100, 35th edition, guidelines, which do not provide ertapenem interpretive criteria for *A. baumannii*. Therefore, ertapenem MICs were not interpreted and were not considered in the assessment of carbapenem resistance. To clarify this point, we have specified the CLSI version used in the Materials and Methods section and revised the Results section accordingly.

Materials and Methods

The original text:

"Minimum inhibitory concentrations (MICs) were interpreted in accordance with the Clinical and Laboratory Standards Institute (CLSI) M100 guidelines [22]."

has been revised to:

"Minimum inhibitory concentrations (MICs) were interpreted in accordance with the Clinical and Laboratory Standards Institute (CLSI) M100, 35th edition, guidelines [22]."

Results

The original text:

"All isolates were resistant to all tested carbapenems, including imipenem, meropenem, and doripenem."

has been revised to:

"All isolates were resistant to imipenem, meropenem, and doripenem. Ertapenem was included in the Sensititre antimicrobial panel; however, the CLSI M100, 35th edition, does not provide ertapenem interpretive breakpoints for *A. baumannii*. Therefore, ertapenem MICs were not interpreted and were not considered in the assessment of carbapenem resistance."

Lines 206-208 – Complete genome assemblies were generated for seven isolates, which were chosen based on the resistance to all tested antibiotics. As there were 8 isolates that were resistant to all antimicrobials (Lines 198-9), why did the authors exclude one isolate? What was the criteria?

We thank the reviewer for identifying this inconsistency. The statement in the original manuscript was inaccurate. The seven isolates selected for complete genome sequencing were not chosen on the basis of resistance to all tested antibiotics. This is also evident from the antimicrobial susceptibility profiles shown in Figure 2. Rather, the isolates were selected to represent the diversity of *bla*$_{NDM}$ genotypes identified in the collection, including *bla*$_{NDM-1}$-positive, *bla*$_{NDM-5}$-positive, and *bla*$_{NDM}$-negative isolates. We have revised the Materials and Methods and Results sections accordingly.

Materials and Methods

The original text:

"To improve the resolution of comparative genomic analyses, long-read sequencing was performed for seven isolates selected based on their resistance to all tested antibiotics."

has been revised to:

"To improve the resolution of comparative genomic analyses, long-read sequencing was performed for seven isolates selected to represent the diversity of *bla*$_{NDM}$ genotypes identified in the collection, including one *bla*$_{NDM-1}$-positive, two *bla*$_{NDM-5}$-positive, and four *bla*$_{NDM}$-negative isolates."

Results

The original text:

"Complete genome assemblies were generated for seven isolates (AB0001–AB0007) using combined Illumina and Oxford Nanopore Technologies sequencing. These isolates were selected due to resistance to all tested antibiotics."

has been revised to:

"Complete genome assemblies were generated for seven isolates (AB0001–AB0007) using combined Illumina and Oxford Nanopore Technologies sequencing. The isolates were selected to represent the diversity of *bla*$_{NDM}$ genotypes identified in the collection, including one *bla*$_{NDM-1}$-positive isolate, two *bla*$_{NDM-5}$-positive isolates, and four isolates lacking *bla*$_{NDM}$ genes."

Lines 263-264 – NDM-5 was detected in ST2 isolates. How many isolates carried NDM-5? This information is never written in the whole text.

We thank the reviewer for this helpful comment. We agree that the number of *bla*$_{NDM-5}$-positive isolates should be explicitly stated when describing its distribution. Accordingly, we have revised the Results section to indicate that *bla*$_{NDM-5}$ was detected in 17 isolates, all belonging to ST2.

The original text:

"In addition to the OXA-type carbapenemase genes, *bla*$_{NDM-5}$ was detected in ST2 isolates distributed across two distinct phylogenetic groups and multiple regions of Thailand."

has been revised to:

"In addition to the OXA-type carbapenemase genes, *bla*$_{NDM-5}$ was detected in 17 ST2 isolates distributed across two distinct phylogenetic groups and multiple regions of Thailand."

Lines 276-277 – How does the authors explain that the ST164 isolate carry the ant(3'')-IIa gene, but remain susceptible to aminoglycosides, especially to gentamicin? At least a short (possible) justification should be included in the discussion.

We thank the reviewer for this insightful comment. The ST164 isolate carried ant(3")-IIa but remained susceptible to all tested aminoglycosides. This observation is not unexpected because ANT(3")-IIa primarily confers resistance to streptomycin and spectinomycin and is not typically associated with resistance to clinically relevant aminoglycosides such as gentamicin, amikacin, or netilmicin. Therefore, the susceptibility phenotype observed in the ST164 isolate is consistent with the known substrate specificity of this enzyme. To clarify this point, we have revised the text to indicate that the observed phenotype is consistent with the limited substrate specificity of ANT(3")-IIa.

The original text:

"The ST164 isolate carried only ant(3")-IIa and remained susceptible to all tested aminoglycosides."

has been revised to:

"The ST164 isolate carried only ant(3")-IIa and remained susceptible to all tested aminoglycosides, consistent with the limited substrate spectrum of ANT(3")-IIa."

Lines 284-300 – The authors mention that the NDM-5 gene is embedded into transposon Tn125, which has flanking ISAba125 elements. However, this is not reflected in figure 3, which has ISAba125 only represented on one side and, as I understand, only the IR of the IS is represented. The ISs are also missing from the two isolates that the authors use to compare with their isolate. Are they absent from the isolates?

A chromosomal location is identified for the Ab0003 isolate and in several parts of the text it is suggested the horizontal acquisition of the NDM-5 gene and associated MGE (for instance, line 404, 422, ...). As WGS was performed, did the authors detect the target site duplication that is typical of transposition and that would strengthen this idea of dissemination?

We thank the reviewer for this important observation. Upon re-examination of the *bla*$_{NDM-5}$ genetic context, we agree that Figure 3 does not depict a canonical Tn125 structure flanked by two complete ISAba125 elements. In our isolates, *bla*$_{NDM-5}$ was associated with a mobile genetic element containing a class 1 integron (*intI1*, *dfrA17*, *aadA5*, *qacEΔ1*, and *sul1*), together with downstream mobile genetic elements including an IS91 family transposase, *dabD*, *trpF*, *ble*$_{MBL}$, *bla*$_{NDM-5}$, and IS*Aba125*. This genetic arrangement has previously been described in *A. baumannii* isolates in Thailand. To improve the accuracy of the genetic structure description, we have revised the manuscript throughout and replaced the term "*bla*$_{NDM-5}$–harboring integron" with "*bla*$_{NDM-5}$–harboring mobile genetic element (MGE)," where appropriate.

In addition, we revised the manuscript to avoid implying that *bla*$_{NDM-5}$ was embedded within a canonical Tn125 transposon flanked by IS*Aba125* elements.

Regarding target site duplications, we examined the chromosomal flanking regions of the *bla*$_{NDM-5}$–harboring MGE but did not identify clear target site duplications that would support a recent transposition event. Therefore, we have avoided making definitive conclusions regarding the precise mechanism of acquisition and instead describe the structure as a *bla*$_{NDM-5}$–harboring MGE.

Change in manuscript text:

Abstract

The original text:

"In particular, *bla*$_{NDM-5}$ was located within a highly conserved class 1 integron flanked by IS*Aba125* and was detected across multiple ST2 sublineages. This integron was distributed among distinct phylogenetic backgrounds and across multiple geographic regions throughout

Thailand, with phylogenetic evidence supporting widespread horizontal dissemination followed by local clonal expansion."

has been revised to:

"In particular, *bla*$_{NDM-5}$ was located within a highly conserved MGE associated with a class 1 integron, IS91 family transposase and IS*Aba125* and was detected across multiple ST2 sublineages. This MGE was distributed across multiple geographic regions and phylogenetic lineages in Thailand, with evidence suggesting widespread horizontal dissemination followed by local clonal expansion."

This revision was made to more accurately describe the genetic context of *bla*$_{NDM-5}$ and to avoid implying that the entire *bla*$_{NDM-5}$-containing structure represents a class 1 integron or a canonical Tn125 transposon flanked by ISAba125 elements.

Introduction

The original text:

"The mobility of *bla*$_{NDM}$ genes is often mediated by transposons such as Tn125 and class 1 integrons, which facilitate the rapid dissemination of these genes across bacterial species and geographic regions [11–15] "

has been revised to:

"The mobility of *bla*$_{NDM}$ genes is often associated with transposons, insertion sequences, and class 1 integrons, which facilitate the rapid dissemination of these genes across bacterial species and geographic regions [11–15]"

This revision was made to provide a more accurate and general description of the mobile genetic elements involved in *bla*$_{NDM}$ dissemination and to avoid implying that all *bla*$_{NDM}$-carrying structures are mediated by canonical Tn125 transposons.

Results

The original result title text:

"Characterization of the *bla*$_{NDM-5}$–harboring integrons"

has been revised to:

"Characterization of the *bla*$_{NDM-5}$ MGE"

The original text:

"Further characterization of the *bla*$_{NDM-5}$–harboring region identified a class 1 integron that was embedded within the composite transposon Tn*125* and was characterized by the presence of *intI1* and flanking IS*Aba125* elements. In addition to *bla*$_{NDM-5}$, this integron contained multiple antimicrobial resistance genes, including *dfrA17* (trimethoprim resistance), *aadA5* (aminoglycoside resistance), *sul1* (sulfonamide resistance), *ble*$_{MBL}$ (bleomycin resistance), and the disinfectant tolerance gene *qacEΔ1*. This *bla*$_{NDM-5}$–harboring integron has been previously described in *A. baumannii* isolates in Thailand [11]"

has been revised to:

"Further characterization of the *bla*$_{NDM-5}$ MGE identified a class 1 integron containing *intI1*, *dfrA17*, *aadA5*, *qacEΔ1*, and *sul1*, followed by an IS91 family transposase, *dabD*, *trpF*, *ble*$_{MBL}$, *bla*$_{NDM-5}$, and IS*Aba125*. This genetic arrangement has previously been described in *A. baumannii* isolates in Thailand [11]."

This revision was made to more accurately describe the organization of the *bla*$_{NDM-5}$-containing region and to avoid implying the presence of a canonical Tn125 transposon flanked by ISAba125 elements. The revised text distinguishes the class 1 integron from the downstream mobile genetic elements associated with *bla*$_{NDM-5}$.

The description of the associated resistance genes was moved to the Discussion section, as follows:

"Detailed analysis revealed that *bla*$_{NDM-5}$ was embedded within a highly conserved MGE, with additional resistance genes including *dfrA17* (trimethoprim resistance), *aadA5* (aminoglycoside resistance), *sul1* (sulfonamide resistance), *ble*$_{MBL}$ (bleomycin resistance), and *qacEΔ1* (disinfectant tolerance). This *bla*$_{NDM-5}$ MGE has been previously reported in *A. baumannii* isolates from clinical specimens and hospital environments in Thailand collected between 2020 and 2021 [11]."

In addition to the revisions described above, we have made corresponding terminology changes throughout the manuscript and replaced the term "*bla*$_{NDM-5}$–harboring integron" with "*bla*$_{NDM-5}$–harboring mobile genetic element (MGE)" where appropriate to ensure consistency and accurately reflect the genetic structure identified in this study.

Lines 312-328 and figure 4—the inclusion of isolates other than the 55 CRAB was never mentioned in the methods and a bunch of results appear with a comparison with isolates from a previous study. This needs to be included in the methodology section.

We thank the reviewer for this helpful comment. We agree that the source and inclusion criteria for the comparison isolates used in Figure 4 were not sufficiently described in the Methods section. To address this issue, we have revised the Materials and Methods section to describe the inclusion of previously reported *bla*$_{NDM-5}$-positive A. baumannii isolates used for comparative phylogenetic analysis.

In Method: Phylogenetic analysis and visualization

The original text:

"Core genome SNPs, including nucleotide substitutions as well as small insertions and deletions, were identified using Snippy v4.6.0. *A. baumannii* strain ACICU (GenBank accession NC_010611.1) was used as the reference genome. Maximum-likelihood phylogenetic analysis was performed using IQ-TREE with the GTR+G nucleotide substitution model and 1,000 bootstrap replicates [32]. Clades with bootstrap support greater than 60% under maximum-likelihood analysis were considered strongly supported. The resulting phylogenetic tree was visualized and annotated using the Interactive Tree of Life (iTOL) v7 web tool [33]."

has been revised to:

"Core genome SNPs, including nucleotide substitutions as well as small insertions and deletions, were identified using Snippy v4.6.0. *A. baumannii* strain ACICU (GenBank accession NC_010611.1) was used as the reference genome. Maximum-likelihood phylogenetic analysis was performed using IQ-TREE with the GTR+G nucleotide substitution model and 1,000 bootstrap replicates [32]. Clades with bootstrap support greater than 60% under maximum-likelihood analysis were considered strongly supported. For comparative analysis of the *bla*$_{NDM-5}$ MGE, previously reported *bla*$_{NDM-5}$-positive *A. baumannii* genomes from Thailand [11] were retrieved from public databases and included in a dedicated phylogenetic analysis of *bla*$_{NDM-5}$-positive isolates. The resulting phylogenetic tree was visualized and annotated using the Interactive Tree of Life (iTOL) v7 web tool [33]."

Line 347—Here applies the same as before for NDM-5. Did the authors detect the typical target site duplication characteristic of transposition?

We thank the reviewer for this comment. Because the genome of AB0005 was fully resolved, we were able to confirm that the *bla*$_{NDM-1}$-containing Tn*125* structure is located on the chromosome. However, we did not identify clear target site duplications flanking the element. Therefore, while the chromosomal location of Tn*125* could be determined, we cannot establish whether the element was acquired through a recent transposition event. Accordingly, the manuscript states only that “Because the genome of AB0005 was fully resolved, further analysis confirmed that Tn*125* is chromosomally located in this isolate,” and does not make definitive conclusions regarding the mechanism by which the element was acquired.

Minor comments

Line 125 – Add the brand of the nephelometer.

We thank the reviewer for this comment. The manufacturer of the nephelometer used for inoculum preparation (Thermo Fisher Scientific, USA) has now been added to the Materials and Methods section.

Line 321– is “one isolate from the current study” or two isolates?

We thank the reviewer for identifying this error. The text has been corrected to indicate that two isolates from the current study, rather than one, were present in the Northern subclade.

The original text:

“...one isolate from the current study...”

has been revised to:

“...two isolates from the current study...”
