# Supplementary material for: Genomic surveillance of carbapenem-resistant Acinetobacter baumannii from bloodstream infections in Thailand reveals widespread dissemination of blaNDM–harboring mobile genetic elements
Source: PLoS One. 2026 Jul 13;21(7):e0353488. doi: 10.1371/journal.pone.0353488 (PMC13362128; doi:10.1371/journal.pone.0353488)
Supplement: Peer review history

# Peer review: Decision Letter 0

**DOI:** 10.1371/journal.pone.0353488.r001

**Authors:** Mustafa Sadek

18 Jun 2026

PONE-D-26-07266

Genomic surveillance of carbapenem-resistant Acinetobacter baumannii from bloodstream infections in Thailand reveals widespread dissemination of blaNDM–harboring mobile genetic elements

PLOS One

Dear Dr. Premsuriya,

Thank you for submitting your manuscript to PLOS ONE. After careful consideration, we feel that it has merit but does not fully meet PLOS ONE’s publication criteria as it currently stands. Therefore, we invite you to submit a revised version of the manuscript that addresses the points raised during the review process.

Please submit your revised manuscript by Aug 02 2026 11:59PM. If you will need more time than this to complete your revisions, please reply to this message or contact the journal office at plosone@plos.org. When you're ready to submit your revision, log on to https://www.editorialmanager.com/pone/ and select the 'Submissions Needing Revision' folder to locate your manuscript file.

Please include the following items when submitting your revised manuscript:

- A letter that responds to each point raised by the academic editor and reviewer(s). You should upload this letter as a separate file labeled 'Response to Reviewers'.
- A marked-up copy of your manuscript that highlights changes made to the original version. You should upload this as a separate file labeled 'Revised Manuscript with Track Changes'.
- An unmarked version of your revised paper without tracked changes. You should upload this as a separate file labeled 'Manuscript'.

If you would like to make changes to your financial disclosure, please include your updated statement in your cover letter. Guidelines for resubmitting your figure files are available below the reviewer comments at the end of this letter.

If applicable, we recommend that you deposit your laboratory protocols in protocols.io to enhance the reproducibility of your results. Protocols.io assigns your protocol its own identifier (DOI) so that it can be cited independently in the future. For instructions see: https://journals.plos.org/plosone/s/submission-guidelines#loc-laboratory-protocols. Additionally, PLOS ONE offers an option for publishing peer-reviewed Lab Protocol articles, which describe protocols hosted on protocols.io. Read more information on sharing protocols at https://plos.org/protocols?utm_medium=editorial-email&utm_source=authorletters&utm_campaign=protocols.

As the corresponding author, your ORCID iD is verified in the submission system and will appear in the published article. PLOS supports the use of ORCID, and we encourage all coauthors to register for an ORCID iD and use it as well. Please encourage your coauthors to verify their ORCID iD within the submission system before final acceptance, as unverified ORCID iDs will not appear in the published article. *Only*  the individual author can complete the verification step; PLOS staff *cannot*  verify ORCID iDs on behalf of authors.

We look forward to receiving your revised manuscript.

Kind regards,

Mustafa Sadek

Academic Editor

PLOS One

**Journal Requirements:**

1. When submitting your revision, we need you to address these additional requirements.

Please ensure that your manuscript meets PLOS ONE's style requirements, including those for file naming. The PLOS ONE style templates can be found at

https://journals.plos.org/plosone/s/file?id=wjVg/PLOSOne_formatting_sample_main_body.pdf and

https://journals.plos.org/plosone/s/file?id=ba62/PLOSOne_formatting_sample_title_authors_affiliations.pdf

2. Please note that your Data Availability Statement is currently missing the direct link to access each database. If your manuscript is accepted for publication, you will be asked to provide these details on a very short timeline. We therefore suggest that you provide this information now, though we will not hold up the peer review process if you are unable.

3. We note that there is identifying data in the Supporting Information file <S1 Table>. Due to the inclusion of these potentially identifying data, we have removed this file from your file inventory. Prior to sharing human research participant data, authors should consult with an ethics committee to ensure data are shared in accordance with participant consent and all applicable local laws.

Data sharing should never compromise participant privacy. It is therefore not appropriate to publicly share personally identifiable data on human research participants. The following are examples of data that should not be shared:

-Name, initials, physical address

-Ages more specific than whole numbers

-Internet protocol (IP) address

-Specific dates (birth dates, death dates, examination dates, etc.)

-Contact information such as phone number or email address

-Location data

-ID numbers that seem specific (long numbers, include initials, titled “Hospital ID”) rather than random (small numbers in numerical order)

Data that are not directly identifying may also be inappropriate to share, as in combination they can become identifying. For example, data collected from a small group of participants, vulnerable populations, or private groups should not be shared if they involve indirect identifiers (such as sex, ethnicity, location, etc.) that may risk the identification of study participants.

Additional guidance on preparing raw data for publication can be found in our Data Policy (https://journals.plos.org/plosone/s/data-availability#loc-human-research-participant-data-and-other-sensitive-data) and in the following article: http://www.bmj.com/content/340/bmj.c181.long.

Please remove or anonymize all personal information (<specific identifying information in file to be removed>), ensure that the data shared are in accordance with participant consent, and re-upload a fully anonymized data set. Please note that spreadsheet columns with personal information must be removed and not hidden as all hidden columns will appear in the published file.

4. Your ethics statement should only appear in the Methods section of your manuscript. If your ethics statement is written in any section besides the Methods, please move it to the Methods section and delete it from any other section. Please ensure that your ethics statement is included in your manuscript, as the ethics statement entered into the online submission form will not be published alongside your manuscript.

5. Please upload a new copy of Figure 2, as the detail is not clear. Please follow the link for more information:  https://journals.plos.org/plosone/s/figures

6. We note that Figures 1a and 1b in your submission contain map images which may be copyrighted. All PLOS content is published under the Creative Commons Attribution License (CC BY 4.0), which means that the manuscript, images, and Supporting Information files will be freely available online, and any third party is permitted to access, download, copy, distribute, and use these materials in any way, even commercially, with proper attribution. For these reasons, we cannot publish previously copyrighted maps or satellite images created using proprietary data, such as Google software (Google Maps, Street View, and Earth). For more information, see our copyright guidelines: http://journals.plos.org/plosone/s/licenses-and-copyright.

We require you to either present written permission from the copyright holder to publish these figures specifically under the CC BY 4.0 license, or remove the figures from your submission:

a. You may seek permission from the original copyright holder of Figures 1a and 1b to publish the content specifically under the CC BY 4.0 license.

We recommend that you contact the original copyright holder with the Content Permission Form (http://journals.plos.org/plosone/s/file?id=7c09/content-permission-form.pdf) and the following text:

“I request permission for the open-access journal PLOS ONE to publish XXX under the Creative Commons Attribution License (CCAL) CC BY 4.0 (http://creativecommons.org/licenses/by/4.0/). Please be aware that this license allows unrestricted use and distribution, even commercially, by third parties. Please reply and provide explicit written permission to publish XXX under a CC BY license and complete the attached form.”

Please upload the completed Content Permission Form or other proof of granted permissions as an "Other" file with your submission.

In the figure caption of the copyrighted figure, please include the following text: “Reprinted from [ref] under a CC BY license, with permission from [name of publisher], original copyright [original copyright year].”

b. If you are unable to obtain permission from the original copyright holder to publish these figures under the CC BY 4.0 license or if the copyright holder’s requirements are incompatible with the CC BY 4.0 license, please either i) remove the figure or ii) supply a replacement figure that complies with the CC BY 4.0 license. Please check copyright information on all replacement figures and update the figure caption with source information. If applicable, please specify in the figure caption text when a figure is similar but not identical to the original image and is therefore for illustrative purposes only.

The following resources for replacing copyrighted map figures may be helpful:

USGS National Map Viewer (public domain): http://viewer.nationalmap.gov/viewer/

The Gateway to Astronaut Photography of Earth (public domain): http://eol.jsc.nasa.gov/sseop/clickmap/

Maps at the CIA (public domain): https://www.cia.gov/library/publications/the-world-factbook/index.html and https://www.cia.gov/library/publications/cia-maps-publications/index.html

NASA Earth Observatory (public domain): http://earthobservatory.nasa.gov/

Landsat: http://landsat.visibleearth.nasa.gov/

USGS EROS (Earth Resources Observatory and Science (EROS) Center) (public domain): http://eros.usgs.gov/#

Natural Earth (public domain): http://www.naturalearthdata.com/

7. Please include captions for your Supporting Information files at the end of your manuscript, and update any in-text citations to match accordingly. Please see our Supporting Information guidelines for more information: http://journals.plos.org/plosone/s/supporting-information.

8. If the reviewer comments include a recommendation to cite specific previously published works, please review and evaluate these publications to determine whether they are relevant and should be cited. There is no requirement to cite these works unless the editor has indicated otherwise.

[Note: HTML markup is below. Please do not edit.]

Reviewers' comments:

Reviewer's Responses to Questions

**Comments to the Author**

1. Is the manuscript technically sound, and do the data support the conclusions?

The manuscript must describe a technically sound piece of scientific research with data that supports the conclusions. Experiments must have been conducted rigorously, with appropriate controls, replication, and sample sizes. The conclusions must be drawn appropriately based on the data presented.

Reviewer #1: Yes

Reviewer #2: Yes

Reviewer #3: Yes

**********

2. Has the statistical analysis been performed appropriately and rigorously?

Reviewer #1: Yes

Reviewer #2: No

Reviewer #3: N/A

**********

3. Have the authors made all data underlying the findings in their manuscript fully available?

The PLOS Data policy requires authors to make all data underlying the findings described in their manuscript fully available without restriction, with rare exception (please refer to the Data Availability Statement in the manuscript PDF file). The data should be provided as part of the manuscript or its supporting information, or deposited to a public repository. For example, in addition to summary statistics, the data points behind means, medians and variance measures should be available. If there are restrictions on publicly sharing data—e.g. participant privacy or use of data from a third party—those must be specified.

Reviewer #1: Yes

Reviewer #2: Yes

Reviewer #3: Yes

**********

4. Is the manuscript presented in an intelligible fashion and written in standard English?

PLOS ONE does not copyedit accepted manuscripts, so the language in submitted articles must be clear, correct, and unambiguous. Any typographical or grammatical errors should be corrected at revision, so please note any specific errors here.

Reviewer #1: Yes

Reviewer #2: Yes

Reviewer #3: Yes

**********

5. Review Comments to the Author

Please use the space provided to explain your answers to the questions above. You may also include additional comments for the author, including concerns about dual publication, research ethics, or publication ethics. (Please upload your review as an attachment if it exceeds 20,000 characters)

**Reviewer #1:** The study is good and the authors have demonstrated a good grasp of the problem they tried to address. The manuscript is well writted and technically sound. The authors should provide details of how the bacteria were subcultured prior to biochemical identification and genomic studies.

**Reviewer #2:**  The study was well researched and findings well presented. Apart from lack of statistical backing, every aspect of the write is impeccably written. The findings made represent the current situation and calls for a

continuation of genomic surveillance to monitor the emergence and dissemination of

blaNDM-producing CRAB and also to strengthen infection prevention and antimicrobial

resistance control efforts in hospital settings. This is a very important call as it represents a critical need in our global health system

**Reviewer #3:** Antibiotic resistance is a major health threat and Acinetobacter baumannii is one of the pathogens included in the critical level of the WHO priority list. Unravelling resistance determinants and distribution patterns of resistant isolates is extremely important to tackle bacterial infections due to multidrug resistant species. In this study, the authors explore the resistant genes and associated mobile genetic elements associated with carbapenem-resistant A. baumannii from bloodstream infections in Thailand. The authors have done a nationwide study, collecting isolates from multiple regions. However, only 41 isolates (+14 from other sources) were taken into consideration, which I find a little low to represent one country and see it as the main limitation of the study. Another weakness of the study as it is, is that the authors de not fully explore the information obtained by WGS (for instance, regarding detection of transposition associated with NDM-carrying mobile genetic elements).

Specific comments are presented below:

Lines 59-61 -OXA-51-like variants is only related to carbapenem resistance when associated with insertion sequences such as ISAba1 or ISAba125, which work as the promotor for the gene expression. This fact is never mentioned during the text, including during the analysis of the results of the genes carried by the studied isolates. WGS was performed and the presence or absence of such insertion sequences was not acknowledged (lines 258-262).

Lines 114-115 – “Species identification was reconfirmed…”. What was the original identification method?

Lines 176-178 – The authors chose to analyse the A. baumannii bloodstream isolates, which only account for 6.26 % of the total A. baumannii isolates they had available. Why choosing this? Nothing is written to justify this choice.

Lines 192-194 – Carbapenems, cephalosporins, aminoglycosides, fluoroquinolones are antimicrobial classes, but cotrimoxazole and colistin no.

Lines 194-195 – “All isolates were resistant to all tested carbapenems, including imipenem, meropenem, and doripenem”. What about ertapenem? Either the isolates are resistant to all tested carbepenems, which include ertapenem, or are not resistant to all.

Lines 206-208 – Complete genome assemblies were generated for seven isolates, which were chosen based on the resistance to all tested antibiotics. As there were 8 isolates that were resistant to all antimicrobials (Lines 198-9), why did the authors exclude one isolate? What was the criteria?

Lines 263-264 – NDM-5 was detected in ST2 isolates. How many isolates carried NDM-5? This information is never written in the whole text.

Lines 276-277 – How does the authors explain that the ST164 isolate carry the ant(3’’)-IIa gene, but remain susceptible to aminoglycosides, especially to gentamicin? At least a short (possible) justification should be included in the discussion.

Lines 284-300 – The authors mention that the NDM-5 gene is embedded into transposon Tn125, which has flanking ISAba125 elements. However, this is not reflected in figure 3, which has ISAba125 only represented on one side and, as I understand, only the IR of the IS is represented. The ISs are also missing from the two isolates that the authors use to compare with their isolate. Are they absent from the isolates?

A chromosomal location is identified for the Ab0003 isolate and in several parts of the text it is suggested the horizontal acquisition of the NDM-5 gene and associated MGE (for instance, line 404, 422, …). As WGS was performed, did the authors detect the target site duplication that is typical of transposition and that would strengthen this idea of dissemination?

Lines 312-328 and figure 4 – the inclusion of isolates other than the 55 CRAB was never mentioned in the methods and a bunch of results appear with comparison with isolates from a previous study. This needs to be included in the methodology section.

Line 347 – Here applies the same as before for NDM-5. Did the authors detect the typical target site duplication characteristic of transposition?

Minor comments

Line 125 – Add the brand of the nephelometer.

Line 321– is “one isolate from the current study” or two isolates?

**********

6. PLOS authors have the option to publish the peer review history of their article (what does this mean?). If published, this will include your full peer review and any attached files.

If you choose “no”, your identity will remain anonymous but your review may still be made public.

**Do you want your identity to be public for this peer review?**  For information about this choice, including consent withdrawal, please see our Privacy Policy.

Reviewer #1: **Yes:** Ayodele Oluwaseun Ajayi

Reviewer #2: No

Reviewer #3: No

**********

[NOTE: If reviewer comments were submitted as an attachment file, they will be attached to this email and accessible via the submission site. Please log into your account, locate the manuscript record, and check for the action link "View Attachments". If this link does not appear, there are no attachment files.]

To ensure your figures meet our technical requirements, please review our figure guidelines: https://journals.plos.org/plosone/s/figures

You may also use PLOS’s free figure tool, NAAS, to help you prepare publication quality figures: https://journals.plos.org/plosone/s/figures#loc-tools-for-figure-preparation.

NAAS will assess whether your figures meet our technical requirements by comparing each figure against our figure specifications.

# Author comment: Author response to Decision Letter 1

**DOI:** 10.1371/journal.pone.0353488.r002

18 Jun 2026

Responses to Reviewers' comments

Reviewer #1: The study is good and the authors have demonstrated a good grasp of the problem they tried to address. The manuscript is well written and technically sound. The authors should provide details of how the bacteria were subcultured prior to biochemical identification and genomic studies.

We thank the reviewer for the positive evaluation of our study and for this helpful suggestion. Additional details regarding isolate subculturing prior to biochemical identification have been added to the Materials and Methods section.

The original text:

“All isolates were retrieved from glycerol stocks that had been collected as part of routine antimicrobial resistance surveillance and stored at −80°C until analysis. Species identification was reconfirmed using standard biochemical methods according to the Manual of Clinical Microbiology”

Has been revised to:

" All isolates were retrieved from glycerol stocks that had been collected as part of routine antimicrobial resistance surveillance and stored at −80°C until analysis. Prior to biochemical identification, isolates were subcultured on sheep blood agar and incubated at 37°C, overnight. Species identification was reconfirmed using standard biochemical methods according to the Manual of Clinical Microbiology”

Reviewer #2: The study was well researched and findings well presented. Apart from lack of statistical backing, every aspect of the write is impeccably written. The findings made represent the current situation and calls for a continuation of genomic surveillance to monitor the emergence and dissemination of blaNDM-producing CRAB and also to strengthen infection prevention and antimicrobial resistance control efforts in hospital settings. This is a very important call as it represents a critical need in our global health system

We thank the reviewer for the positive evaluation of our study and for recognizing the importance of continued genomic surveillance of carbapenem-resistant Acinetobacter baumannii. We appreciate the reviewer’s support for our conclusions regarding the need for ongoing monitoring of blaNDM-producing CRAB and for strengthened infection prevention and antimicrobial resistance control efforts.

Reviewer #3: Antibiotic resistance is a major health threat and Acinetobacter baumannii is one of the pathogens included in the critical level of the WHO priority list. Unravelling resistance determinants and distribution patterns of resistant isolates is extremely important to tackle bacterial infections due to multidrug resistant species. In this study, the authors explore the resistant genes and associated mobile genetic elements associated with carbapenem-resistant A. baumannii from bloodstream infections in Thailand. The authors have done a nationwide study, collecting isolates from multiple regions. However, only 41 isolates (+14 from other sources) were taken into consideration, which I find a little low to represent one country and see it as the main limitation of the study. Another weakness of the study as it is, is that the authors de not fully explore the information obtained by WGS (for instance, regarding detection of transposition associated with NDM-carrying mobile genetic elements).

We thank the reviewer for the thoughtful evaluation of our study and for recognizing the importance of investigating resistance determinants and mobile genetic elements among carbapenem-resistant Acinetobacter baumannii. We acknowledge the limitations associated with the sample size and have highlighted this point in the Discussion section.

The following sentence has been added to the Discussion:

“While this study provides detailed insights into the genomic epidemiology of CRAB in Thailand, additional studies are required to better characterize the genomic diversity and transmission dynamics of CRAB in Thailand. Although isolates were collected through a nationwide surveillance network and represented multiple geographic regions of Thailand, the relatively limited number of bloodstream CRAB isolates available for genomic analysis may not fully capture the genetic diversity and resistance mechanisms circulating among CRAB populations nationwide. In addition, the study focused exclusively on bloodstream isolates and therefore may not reflect the genomic characteristics of CRAB circulating in other clinical sources or environmental reservoirs.”

Our study focused primarily on bloodstream isolates collected through the national surveillance network, which limited the number of available isolates but allowed us to investigate CRAB associated with severe invasive infections from multiple regions of Thailand.

We also appreciate the reviewer's comment regarding the utilization of WGS data. In this study, we aimed to focus on the characterization and dissemination of blaNDM-associated mobile genetic elements. Accordingly, we performed detailed analyses of blaNDM-5–harboring integrons, blaNDM-1–associated Tn125 transposons, their genomic contexts, and their distribution across phylogenetic lineages and geographic regions. Detailed responses to the specific comments are provided below.

Specific comments

Lines 59-61 -OXA-51-like variants is only related to carbapenem resistance when associated with insertion sequences such as ISAba1 or ISAba125, which work as the promotor for the gene expression. This fact is never mentioned during the text, including during the analysis of the results of the genes carried by the studied isolates. WGS was performed and the presence or absence of such insertion sequences was not acknowledged (lines 258-262).

We thank the reviewer for this important comment. We agree that OXA-51-like carbapenemases are most commonly associated with carbapenem resistance when overexpressed through upstream insertion sequences such as ISAba1.

To address this concern, we revised the Introduction to clarify the role of insertion sequence-mediated overexpression of OXA-51-like carbapenemases. In addition, we examined the upstream genomic regions of blaOXA-402 in all ST16 isolates using the ISfinder database and BLAST searches and found that no ISAba1 or ISAba125 elements were present upstream of the gene. This information has been added to the Results section. We have also updated the Materials and Methods section to describe the approach used to identify insertion sequence elements associated with carbapenemase genes.

Changes in manuscript:

Introduction

The original text:

“Resistance of A. baumannii to carbapenem is most commonly mediated by class D OXA-type carbapenemases, especially blaOXA-23-like, and often in combination with intrinsic blaOXA-51-like variants”

has been revised to:

“Resistance of A. baumannii to carbapenem is most commonly mediated by class D OXA-type carbapenemases, especially blaOXA-23-like [4]. Intrinsic blaOXA-51-like variants may also contribute to carbapenem resistance, particularly when overexpressed through the presence of upstream insertion sequences such as ISAba1 that provide strong promoter activity [5,6].”

Materials and methods

The following text has been added to: Genomic assembly and annotation

“The genomic regions surrounding carbapenemase genes were examined using the ISfinder database and confirmed by BLAST search to identify associated insertion sequence elements [29].”

Results

The original text:

“For ST16, all isolates harbored blaOXA-402 (OXA-51-like beta-lactamase), with some isolates additionally co-harboring blaOXA-23 and/or blaOXA-58.”

has been revised to:

“For ST16, all isolates harbored blaOXA-402 (OXA-51-like beta-lactamase), with some isolates additionally co-harboring blaOXA-23 and/or blaOXA-58. No ISAba1 or ISAba125 elements were detected upstream of OXA-51-like beta-lactamase in any ST16 isolate.”

Lines 114-115 – “Species identification was reconfirmed…”. What was the original identification method?

We thank the reviewer for this comment. The isolates were initially identified as A. baumannii by participating hospitals as part of routine clinical microbiology laboratory procedures before submission to NARST. Species identification was subsequently reconfirmed prior to genomic analysis. We have clarified this information in the Materials and Methods section.

The original text:

“A total of 55 CRAB isolates collected between 2020 and 2024 were included in this study as part of nationwide genomic surveillance conducted through NARST.”

has been revised to:

“A total of 55 CRAB isolates collected between 2020 and 2024 were included in this study as part of nationwide genomic surveillance conducted through NARST. Isolates were initially identified as A. baumannii by participating hospitals using routine clinical microbiology laboratory procedures before submission to NARST.”

Lines 176-178 – The authors chose to analyse the A. baumannii bloodstream isolates, which only account for 6.26 % of the total A. baumannii isolates they had available. Why choosing this? Nothing is written to justify this choice.

We thank the reviewer for this comment. The rationale for focusing on bloodstream isolates was based on their substantial clinical significance and was described in the Introduction.

Specifically, we stated that:

“National surveillance data from 2022 indicated that CRAB was the leading cause of antimicrobial-resistant hospital-origin bloodstream infections. These infections accounted for 51.2% of cases and were associated with mortality rates of up to 60% [20]. While previous studies have described the prevalence of dominant clonal lineages and OXA-type carbapenemases [19–21], comprehensive nationwide genomic data on CRAB bloodstream infections remain limited, particularly with respect to the distribution and genetic context of blaNDM-harboring elements.”

Therefore, we focused our genomic analysis on bloodstream isolates because they represent a severe form of A. baumannii infection associated with high mortality, while also addressing an important knowledge gap regarding the nationwide molecular epidemiology and genomic characteristics of CRAB bloodstream infections in Thailand.

Lines 192-194 – Carbapenems, cephalosporins, aminoglycosides, fluoroquinolones are antimicrobial classes, but cotrimoxazole and colistin no.

We thank the reviewer for this comment. We agree that colistin and trimethoprim–sulfamethoxazole are individual antimicrobial agents. To improve consistency, we have revised the text to classify all antimicrobials according to their respective antimicrobial classes.

Materials and Methods

The original text:

“The antimicrobial panel included penicillins and β-lactam/β-lactamase inhibitor combinations (ampicillin, amoxicillin–clavulanate, ampicillin–sulbactam, piperacillin–tazobactam), cephalosporins (cefuroxime, cefoxitin, cefotaxime, ceftriaxone, ceftazidime, cefepime), carbapenems (imipenem, meropenem, doripenem, ertapenem), aminoglycosides (amikacin, gentamicin, netilmicin), fluoroquinolones (ciprofloxacin, levofloxacin), colistin, and trimethoprim–sulfamethoxazole.”

has been revised to:

“The antimicrobial panel included penicillins and β-lactam/β-lactamase inhibitor combinations (ampicillin, amoxicillin–clavulanate, ampicillin–sulbactam, piperacillin–tazobactam), cephalosporins (cefuroxime, cefoxitin, cefotaxime, ceftriaxone, ceftazidime, cefepime), carbapenems (imipenem, meropenem, doripenem, ertapenem), aminoglycosides (amikacin, gentamicin, netilmicin), fluoroquinolones (ciprofloxacin, levofloxacin), polymyxins (colistin), and folate pathway inhibitors (trimethoprim–sulfamethoxazole).”

Results

The original text:

“The antimicrobial susceptibility profiles of 11 antibiotics, representing six different antimicrobial classes (carbapenems, cephalosporins, aminoglycosides, fluoroquinolones, co-trimoxazole, and colistin), are summarized in S1 Table.”

has been revised to:

“The antimicrobial susceptibility profiles of 11 antibiotics, representing six different antimicrobial classes (carbapenems, cephalosporins, aminoglycosides, fluoroquinolones, polymyxins, and folate pathway inhibitors) are summarized in S1 Table.”

Lines 194-195 – “All isolates were resistant to all tested carbapenems, including imipenem, meropenem, and doripenem”. What about ertapenem? Either the isolates are resistant to all tested carbepenems, which include ertapenem, or are not resistant to all.

We thank the reviewer for this comment. Although ertapenem was included in the antimicrobial susceptibility testing panel, antimicrobial susceptibility results were interpreted according to the Clinical and Laboratory Standards Institute (CLSI) M100, 35th edition, guidelines, which do not provide ertapenem interpretive criteria for A. baumannii. Therefore, ertapenem MICs were not interpreted and were not considered in the assessment of carbapenem resistance. To clarify this point, we have specified the CLSI version used in the Materials and Methods section and revised the Results section accordingly.

Materials and Methods

The original text:

“Minimum inhibitory concentrations (MICs) were interpreted in accordance with the Clinical and Laboratory Standards Institute (CLSI) M100 guidelines [22].”

has been revised to:

“Minimum inhibitory concentrations (MICs) were interpreted in accordance with the Clinical and Laboratory Standards Institute (CLSI) M100, 35th edition, guidelines [22].”

Results

The original text:

“All isolates were resistant to all tested carbapenems, including imipenem, meropenem, and doripenem.”

has been revised to:

“All isolates were resistant to imipenem, meropenem, and doripenem. Ertapenem was included in the Sensititre antimicrobial panel; however, the CLSI M100, 35th edition, does not provide ertapenem interpretive breakpoints for A. baumannii. Therefore, ertapenem MICs were not interpreted and were not considered in the assessment of carbapenem resistance.”

Lines 206-208 – Complete genome assemblies were generated for seven isolates, which were chosen based on the resistance to all tested antibiotics. As there were 8 isolates that were resistant to all antimicrobials (Lines 198-9), why did the authors exclude one isolate? What was the criteria?

We thank the reviewer for identifying this inconsistency. The statement in the original manuscript was inaccurate. The seven isolates selected for complete genome sequencing were not chosen on the basis of resistance to all tested antibiotics. This is also evident from the antimicrobial susceptibility profiles shown in Figure 2. Rather, the isolates were selected to represent the diversity of blaNDM genotypes identified in the collection, including blaNDM-1-positive, blaNDM-5-positive, and blaNDM-negative isolates. We have revised the Materials and Methods and Results sections accordingly.

Materials and Methods

The original text:

“To improve the resolution of comparative genomic analyses, long-read sequencing was performed for seven isolates selected based on their resistance to all tested antibiotics.”

has been revised to:

“To improve the resolution of comparative genomic analyses, long-read sequencing was performed for seven isolates selected to represent the diversity of blaNDM genotypes identified in the collection, including one blaNDM-1-positive, two blaNDM-5-positive, and four blaNDM-negative isolates.”

Results

The original text:

“Complete genome assemblies were generated for seven isolates (AB0001–AB0007) using combined Illumina and Oxford Nanopore Technologies sequencing. These isolates were selected due to resistance to all tested antibiotics.”

has been revised to:

“Complete genome assemblies were generated for seven isolates (AB0001–AB0007) using combined Illumina and Oxford Nanopore Technologies sequencing. The isolates were selected to represent the diversity of blaNDM genotypes identified in the collection, including one blaNDM-1-positive isolate, two blaNDM-5-positive isolates, and four isolates lacking blaNDM genes.”

Lines 263-264 – NDM-5 was detected in ST2 isolates. How many isolates carried NDM-5? This information is never written in the whole text.

We thank the reviewer for this helpful comment. We agree that the number of blaNDM-5-positive isolates should be explicitly stated when describing its distribution. Accordingly, we have revised the Resul

Attachment Submitted filename: Response to Reviewers.pdf

# Editor report: Decision Letter 1

**DOI:** 10.1371/journal.pone.0353488.r003

**Authors:** Mustafa Sadek, Mustafa Sadek

24 Jun 2026

Genomic surveillance of carbapenem-resistant Acinetobacter baumannii from bloodstream infections in Thailand reveals widespread dissemination of blaNDM–harboring mobile genetic elements

PONE-D-26-07266R1

Dear Dr. Premsuriya,

We’re pleased to inform you that your manuscript has been judged scientifically suitable for publication and will be formally accepted for publication once it meets all outstanding technical requirements.

Within one week, you’ll receive an e-mail detailing the required amendments. When these have been addressed, you’ll receive a formal acceptance letter, and your manuscript will be scheduled for publication.

An invoice will be generated when your article is formally accepted. Please note, if your institution has a publishing partnership with PLOS and your article meets the relevant criteria, all or part of your publication costs will be covered. Please make sure your user information is up-to-date by logging into Editorial Manager at Editorial Manager® and clicking the ‘Update My Information' link at the top of the page. For questions related to billing, please contact billing support.

If your institution or institutions have a press office, please notify them about your upcoming paper to help maximize its impact. If they’ll be preparing press materials, please inform our press team as soon as possible -- no later than 48 hours after receiving the formal acceptance. Your manuscript will remain under strict press embargo until 2 pm Eastern Time on the date of publication. For more information, please contact onepress@plos.org.

Kind regards,

Mustafa Sadek

Academic Editor

PLOS One

Additional Editor Comments (optional):

Reviewers' comments:

# Editor report: Acceptance letter

**DOI:** 10.1371/journal.pone.0353488.r004

**Authors:** Mustafa Sadek, Mustafa Sadek

PONE-D-26-07266R1

PLOS One

Dear Dr. Premsuriya,

I'm pleased to inform you that your manuscript has been deemed suitable for publication in PLOS One. Congratulations! Your manuscript is now being handed over to our production team.

At this stage, our production department will prepare your paper for publication. This includes ensuring the following:

* All references, tables, and figures are properly cited

* All relevant supporting information is included in the manuscript submission,

* There are no issues that prevent the paper from being properly typeset

You will receive further instructions from the production team, including instructions on how to review your proof when it is ready. Please keep in mind that we are working through a large volume of accepted articles, so please give us a few days to review your paper and let you know the next and final steps.

Lastly, if your institution or institutions have a press office, please let them know about your upcoming paper now to help maximize its impact. If they'll be preparing press materials, please inform our press team within the next 48 hours. Your manuscript will remain under strict press embargo until 2 pm Eastern Time on the date of publication. For more information, please contact onepress@plos.org.

You will receive an invoice from PLOS for your publication fee after your manuscript has reached the completed accept phase. If you receive an email requesting payment before acceptance or for any other service, this may be a phishing scheme. Learn how to identify phishing emails and protect your accounts at https://explore.plos.org/phishing.

If we can help with anything else, please email us at customercare@plos.org.

Thank you for submitting your work to PLOS ONE and supporting open access.

Kind regards,

PLOS ONE Editorial Office Staff

on behalf of

Dr. Mustafa Sadek

Academic Editor

PLOS One
